# Alterations in the chondrocyte surfaceome in response to pro-inflammatory cytokines

**DOI:** 10.1186/s12860-020-00288-9

**Published:** 2020-06-26

**Authors:** Bernadette Jeremiasse, Csaba Matta, Christopher R. Fellows, David J. Boocock, Julia R. Smith, Susan Liddell, Floris Lafeber, Willem E. van Spil, Ali Mobasheri

**Affiliations:** 1 Department of Rheumatology & Clinical Immunology, University Medical Centre Utrecht, Utrecht, The Netherlands; 2 Department of Anatomy, Histology and Embryology, Faculty of Medicine, University of Debrecen, Debrecen, Hungary; 3 Department of Veterinary Pre-Clinical Sciences, School of Veterinary Science and Medicine, University of Surrey, Guildford, UK; 4 John van Geest Cancer Research Centre, Nottingham Trent University, Nottingham, NG11 8NS UK; 5 Bruker UK Limited, Coventry, UK; 6 Exonate Ltd., Medicity, Thane Road, Nottingham, UK; 7 Research Unit of Medical Imaging, Physics and Technology, Faculty of Medicine, University of Oulu, Oulu, Finland; 8 Department of Regenerative Medicine, State Research Institute Centre for Innovative Medicine, Vilnius, Lithuania; 9 Centre for Sport, Exercise and Osteoarthritis Research Versus Arthritis, Queen’s Medical Centre, Nottingham, UK; 10 Department of Orthopedics, UMC Utrecht, Utrecht, The Netherlands

**Keywords:** Surfaceome, Aminooxy-biotin, Mass spectrometry, Proteomics, Osteoarthritis, Biomarker, Inflammation, Chondrocyte

## Abstract

**Background:**

Chondrocytes are exposed to an inflammatory micro-environment in the extracellular matrix (ECM) of articular cartilage in joint diseases such as osteoarthritis (OA) and rheumatoid arthritis (RA). In OA, degenerative changes and low-grade inflammation within the joint transform the behaviour and metabolism of chondrocytes, disturb the balance between ECM synthesis and degradation, and alter the osmolality and ionic composition of the micro-environment. We hypothesize that chondrocytes adjust their physiology to the inflammatory microenvironment by modulating the expression of cell surface proteins, collectively referred to as the ‘surfaceome’. Therefore, the aim of this study was to characterize the surfaceome of primary equine chondrocytes isolated from healthy joints following exposure to the pro-inflammatory cytokines interleukin-1-beta (IL-1β) and tumour necrosis factor-alpha (TNF-α). We employed combined methodology that we recently developed for investigating the surfaceome in stem cells. Membrane proteins were isolated using an aminooxy-biotinylation technique and analysed by mass spectrometry using high throughput shotgun proteomics. Selected proteins were validated by western blotting.

**Results:**

Amongst the 431 unique cell surface proteins identified, a high percentage of low-abundance proteins, such as ion channels, receptors and transporter molecules were detected. Data are available via ProteomeXchange with identifier PXD014773. A high number of proteins exhibited different expression patterns following chondrocyte stimulation with pro-inflammatory cytokines. Low density lipoprotein related protein 1 (LPR-1), thrombospondin-1 (TSP-1), voltage dependent anion channel (VDAC) 1–2 and annexin A1 were considered to be of special interest and were analysed further by western blotting.

**Conclusions:**

Our results provide, for the first time, a repository for proteomic data on differentially expressed low-abundance membrane proteins on the surface of chondrocytes in response to pro-inflammatory stimuli.

## Background

Osteoarthritis (OA) is one of the most common chronic joint diseases. It is amongst the major causes of pain and disability, affecting more than 25% of the population over 45 years of age [1] and more than 240 million people worldwide [2]. OA is characterized by articular cartilage loss, osteophyte development, subchondral bone changes, and synovial inflammation. It is now widely accepted that inflammatory mediators produced by chondrocytes and synoviocytes such as pro-inflammatory cytokines, nitric oxide (NO), reactive oxygen species (ROS) and matrix degrading enzymes play a role in the initiation and propagation of pathogenic OA processes [3]. Biomechanical stress and joint over-load have been shown to significantly increase the synthesis of pro-inflammatory mediators. The elevated concentration of these mediators during joint inflammation stimulate the gradual deterioration of cartilage, synovial membrane and subchondral bone [3].

There is evidence that cartilage extracellular matrix (ECM) undergoes alterations during OA pathogenesis in terms of glycosaminoglycan (GAG) and water content [4]. These in turn alter the osmolality of the matrix and composition of its ionic milieu [5]. Chondrocytes respond to these changes and attempt to maintain their homeostasis by adjusting the transport of ions across the cell membrane [6] via the complement of transporters and ion channels, collectively referred to as the ‘channelome’ [7, 8]. We therefore hypothesized that chondrocytes adjust their physiology to the inflammatory microenvironment by modulating the expression of these transporters and ion channels during or prior to the onset of symptomatic OA. To capture these alterations, we used proteomics to characterize the surfaceome of primary articular chondrocytes exposed to pro-inflammatory cytokines.

With the use of high throughput proteomics following enrichment of the cell surface proteins, it is possible to identify chondrocyte plasma membrane (PM) proteins under experimental exposure to pro-inflammatory stimuli. The differentially expressed cell surface proteins could potentially be exploited as biomarkers for diagnostic, prognostic, and therapeutic targets of OA.

The aim of this study was to characterize the surfaceome of primary equine articular chondrocytes exposed to a pro-inflammatory micro-environment with a PM protein isolation technique using aminooxy-biotinylation (AOB), providing better enrichment than alternative methods such as Triton X-114 isolation [9]. This technique is based on the principle that surface sialic acid residues on extracellular domains of PM proteins are oxidized and biotinylated, allowing for high percentages of PM proteins to be labelled and enriched. We have successfully applied the same technique to compare the surfaceomes of two closely related cell types, mesenchymal stem cells and cartilage progenitor cells, both relevant to cartilage biology and OA [10]. This methodology is particularly suitable for detecting low-abundance proteins, such as ion channels and transporter molecules, which are hypothesised to be differentially expressed in response to pro-inflammatory cytokines.

## Results

### Experimental workflow

**Fig. 1:**
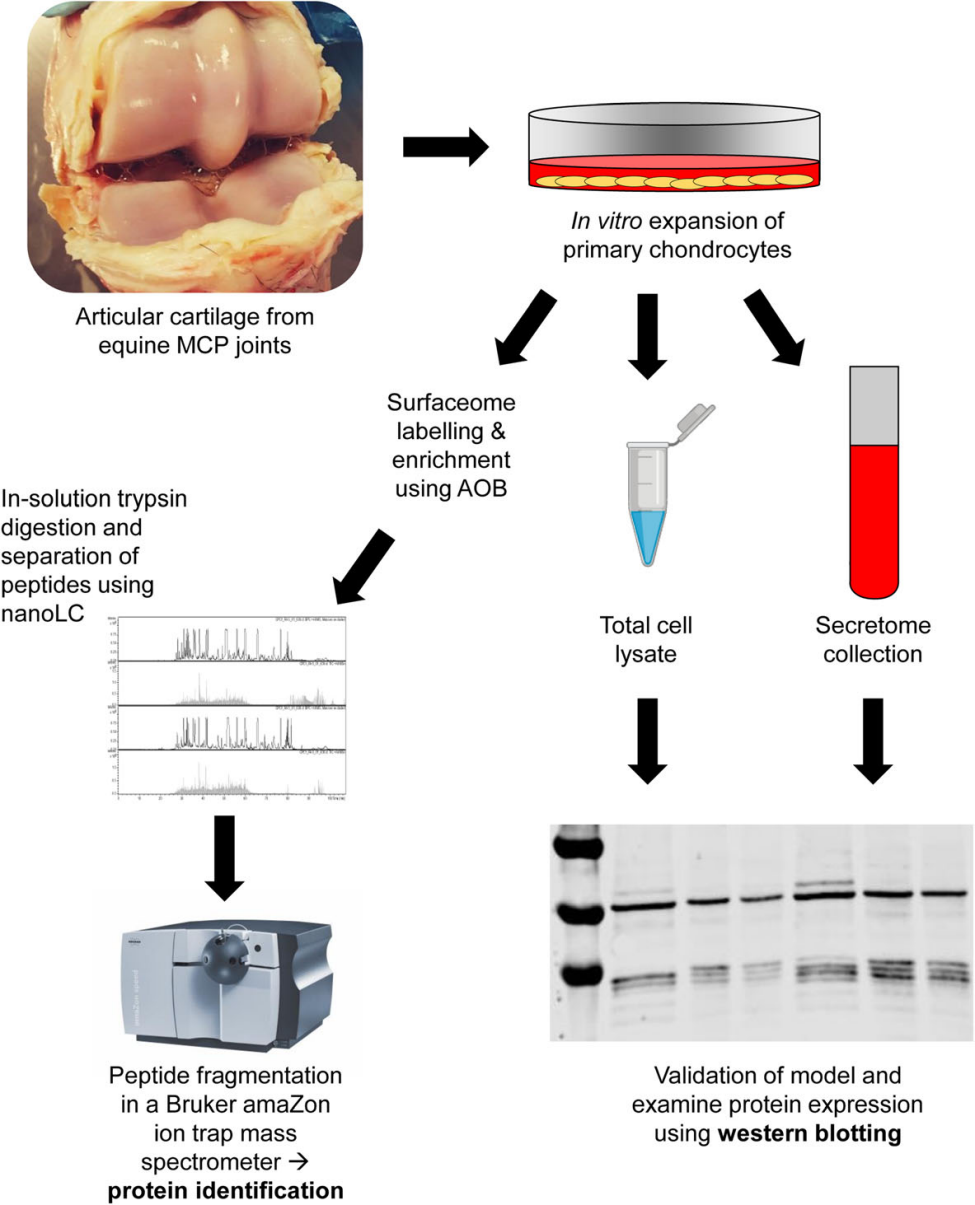
Experimental workflow

The aim of this study was to provide a qualitative description on the surfaceome of primary equine articular chondrocytes following exposure to pro-inflammatory cytokines. After selective enrichment of surface proteins, we generated lists of proteins using shotgun qualitative proteomics (*n* = 2). We then validated some of the proteins qualitatively and also quantified some of the proteins using western blotting. The experimental workflow is presented in Fig. 1.

### Validation of the in vitro inflammatory chondrocyte monolayer model by western blotting

**Fig. 2:**
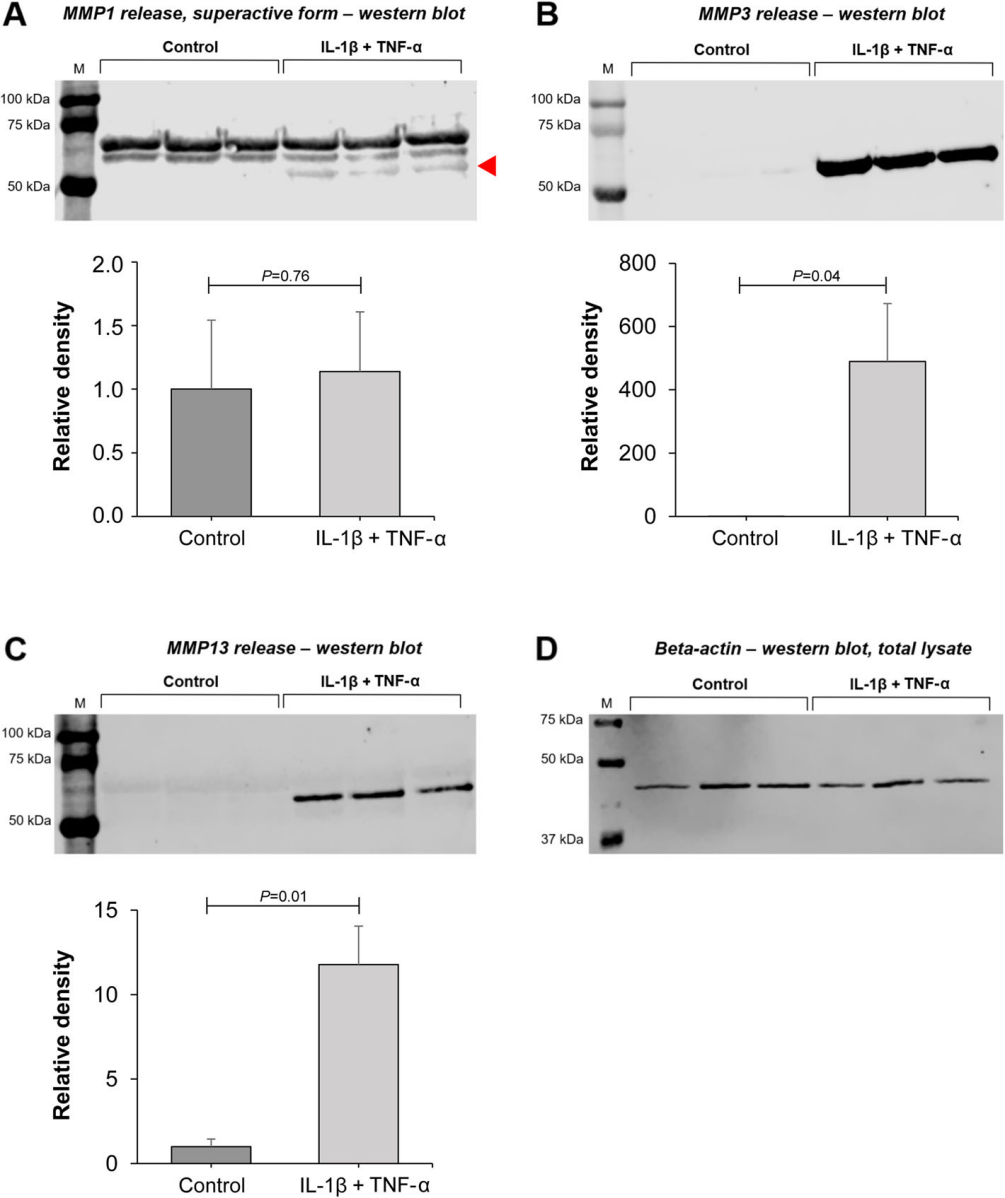
Western blotting and densitometric analysis of MMP-1, − 3, and − 13 release in the secretome of chondrocytes following cytokine treatment (IL-1β + TNF-α, both 10 ng/mL) versus control conditions. **a** Similar MMP-1 release between cytokine-treated chondrocytes vs. control, with the exception of the super-active form of MMP1 (lowest band, indicated by red arrowhead). **b** Increase in MMP-3 release upon cytokine exposure. **c** Increase in MMP-13 release upon cytokine exposure. **d** Beta-actin was measured on western blots of total protein lysates extracted separately from the same samples as **a–c** as loading control in order to correct for cell number, as no reliable housekeeping protein is available for secreted proteins. Measurements from three horses (three biological replicates) were combined to provide final values for each group (mean ± SD)

Western blotting using the secretome of control and cytokine-treated chondrocytes demonstrated a protein band around 53 kDa, corresponding to the predicted molecular mass of matrix metalloproteinase 1 (MMP-1) (Fig. 2a). Densitometry revealed no statistically significant difference in MMP-1 release in response to cytokines (*P* = 0.76). However, the ‘superactive’ cleaved form of MMP-1, represented by the lowest band (marked by arrowhead in Fig. 2a), was present only under inflammatory conditions, indicating an increase in MMP-1 activity [11, 12]. Western blotting of MMP-3 (54 kDa protein band) indicated a very clear, significant difference between untreated and cytokine-treated chondrocytes (*P* = 0.04) (Fig. 2b). MMP-13 release (54 kDa protein band) was also significantly increased by cytokine treatment (*P* = 0.01) (Fig. 2c). In addition, cytokine treatment resulted in a significant decrease of 63 ± 13% in GAG levels in the chondrocyte secretome (*P* = 0.0003) (see Figure S1 in the Supplementary Material, Additional file 1).

### Aminooxy-biotin labelling enriches plasma membrane proteins efficiently

**Fig. 3:**
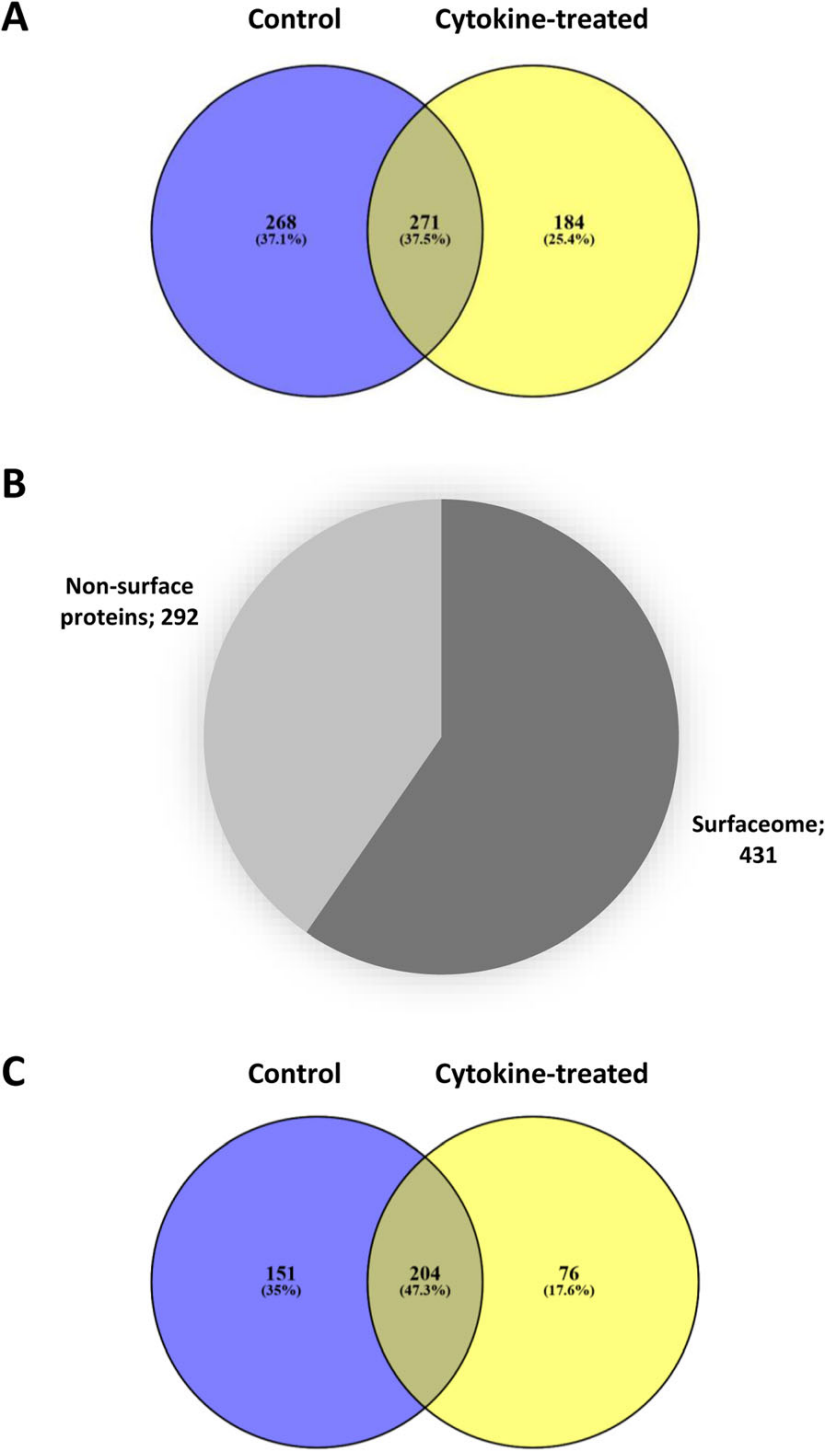
Enrichment of cell surface proteins using the AOB labelling approach. **a** Distribution of the 723 unique proteins identified in this study between untreated (control) and cytokine-exposed equine articular chondrocytes. **b** Of the 723 unique proteins reliably identified in this study, 431 proteins were PM proteins (60%) according to UniProt database entries and gene ontology (GO) annotations. **c** Distribution of the 431 unique cell surface proteins identified in this study between untreated (control) and cytokine-exposed equine articular chondrocytes

Using the AOB labelling approach, a total of 723 unique proteins could be identified reliably (*P* < 0.05). Two hundred sixty-eight proteins (37%) were detected in the control cells only, 184 (25%) were identified in the experimental group only, and 271 (36%) were common between the two groups (Fig. 3a). According to UniProt database entries and Gene Ontology (GO) annotations, 431 proteins were surface proteins (60%) (Fig. 3b; see also Supplementary Tables S1–S6, Additional file 1). The remaining 292 proteins were non-surface proteins. Surface protein enrichment was significantly improved compared to the previously used Triton X-114 phase separation technique, when the ratio of plasma membrane proteins was only 20% (64 out of 315 proteins) [9]. The proportion of surfaceome proteins (60%) was similar to our previous work using the same methodology on cartilage progenitor cells (CPCs) and mesenchymal stem cells (MSCs) [10]. Of the 431 surface proteins, 151 proteins (35%) were detected in control cells only, 76 proteins (18%) were identified in cytokine-treated cells only, and 204 proteins (47%) were common between the two conditions (Fig. 3c), which shows that the distribution of positively identified surface proteins remained quite similar to that of all proteins.

### Classification of plasma membrane proteins reveals various functional subgroups

**Fig. 4:**
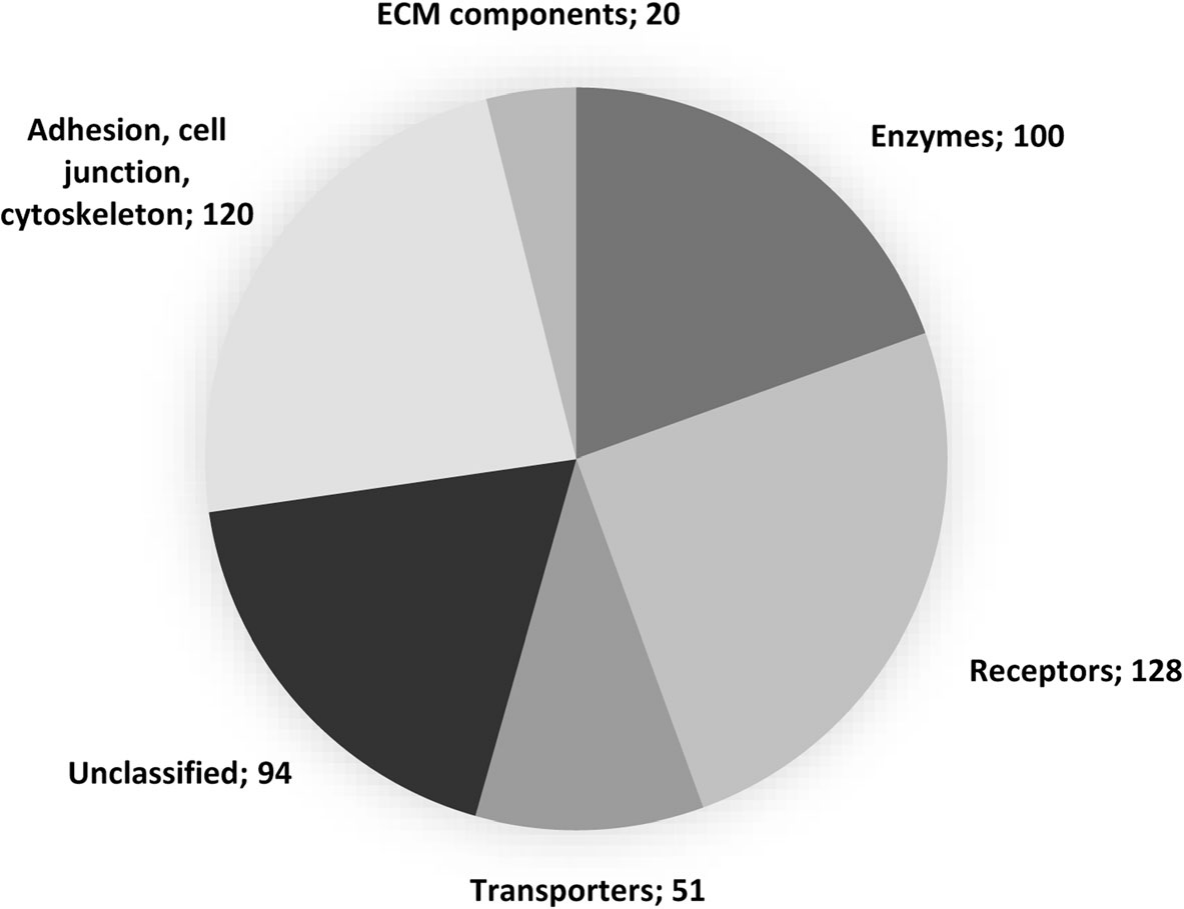
Subcellular distribution of the surface proteins identified in this study. Four hundred thirty-one surface proteins were classified into functional subgroups based on their main function as listed in the UniProt database entries (GO molecular function and/or GO biological process) into the following major functional groups: transporters, receptors, enzymes, extracellular matrix components, proteins involved in cell adhesion and cell junctions, and unclassified proteins. Data are based on labelling performed on chondrocytes obtained from two different horses (2 biological replicates). Note that some proteins appear in more than one category. Numbers in the pie chart represent the actual numbers of proteins in each subgroup

Following the approach used previously on MSCs and CPCs [10], we classified the surfaceome proteins identified in this study into the following major functional groups based on their main GO molecular functions: enzymes, transporters, receptors, proteins mediating cell junctions and adhesion, extracellular matrix components, and unclassified proteins (Fig. 4). Classification was based on the GO annotations (GO molecular function and/or GO biological process) in the UniProt database. During protein classification, we have also picked up known interacting or binding partners of receptor/transporter/enzyme proteins. The full lists of proteins in each subcategory can be found in Supplementary Tables S1–S6 online (Additional file 1). One hundred proteins were classified as enzymes (23%), 128 proteins had receptor roles (30%), 51 proteins were involved in transport processes across the PM (12%), 120 proteins were involved in adhesion, cell-cell or cell-matrix junctions and cytoskeletal organisation (28%), and 20 proteins were structural ECM components (5%). Ninety-four proteins (22%) could not be assigned to one of the subgroups or their function was unknown (Fig. 4).

### Cytokine treatment results in the over-representation of different pathways and protein interactions

When the proteins uniquely identified in either control or cytokine-treated samples were submitted to the Reactome pathway knowledgebase [13], different pathways were found to be over-represented, which suggests that the surface proteins involved in various metabolic pathways could have been influenced by exposure to IL-1β and TNF-α. The most significant pathways in control and cytokine-treated cells based on the unique proteins identified in the surfaceome are listed in Tables 2 and 3. The pathways of interest common in both conditions included ‘Extracellular matrix organisation,’ ‘Integrin cell surface interactions,’ ‘Cell junction organisation,’ ‘Syndecan interactions’ and ‘Cell-cell communication.’ In the control, surface proteins participating in ‘Interleukin-12 signalling,’ ‘L1CAM interactions’ and ‘EPH-ephrin signalling’ pathways were over-represented (Table 2). Following treatment with pro-inflammatory cytokines, ‘Adherens junctions interactions,’ ‘ATF6-alpha activates chaperones,’ ‘Non-integrin membrane-ECM interactions,’ ‘RAB-geranylgeranylation,’ ‘Regulation of Insulin-like Growth Factor (IGF) transport and uptake by Insulin-like Growth Factor Binding Proteins (IGFBPs),’ ‘Transport of inorganic cations/anions and amino acids/oligopeptides,’ ‘Transport of small molecules’ and ‘Vesicle-mediated transport’ pathways were found to be enriched (Table 3). The over-represented pathways in control and cytokine-treated conditions visualised as Voronoi representations (foam tree) are available as Additional files 2 and 3, respectively.

We also submitted our surfaceome dataset to the STRING resource [14] to identify potential protein interactions. We found 339 nodes in the surfaceome proteins in the control, with a fairly high number of edges (992), indicating many interactions between these proteins. The average node degree was 5.85 (see Additional file 4). We found the following biological processes (GO) enriched in the control: localization, vesicle-mediated transport, anatomical structure development, biological adhesion and system development. For GO molecular function, the following terms were significantly enriched: cell adhesion molecule binding, protein binding, transmembrane receptor protein kinase activity and anion binding. As expected, the following cellular components were significantly enriched: cell periphery, plasma membrane, membrane and cell surface. We also looked at the KEGG (Kyoto Encyclopedia of Genes and Genomes) database resource to identify higher level molecular interaction, reaction and relation networks. In terms of KEGG pathways, focal adhesion, cytoskeleton regulation and ECM-receptor interaction were enriched.

Following treatment with pro-inflammatory cytokines, STRING analysis revealed 265 nodes within the surfaceome proteins, also with a high number of edges (829), indicating many interactions between these proteins. The average node degree was 6.26 (see Additional file 5). We found the following biological processes (GO) enriched following treatment with IL-1β and TNF-α: localization, transport, system development and secretion. For GO molecular function, the following terms were significantly enriched: cell adhesion molecule binding, protein binding, integrin binding, signalling receptor binding. Also, as expected, the following cellular components were significantly enriched: cell periphery, plasma membrane, and vesicle. For KEGG pathways, focal adhesion, ECM-receptor interaction, and cancer proteoglycans were enriched.

### Exposure to pro-inflammatory cytokines influences the expression of multiple chondrocyte plasma membrane proteins

The expression of many surface proteins may be influenced by exposure to IL-1β and TNF-α (Tables S1–6 in the Supplementary Material, Additional file 1). Therefore, low density lipoprotein related protein 1 (LPR-1), thrombospondin-1 (TSP-1), voltage dependent anion channel 2 (VDAC2) and annexin A1 were chosen as proteins of special interest, based on literature data supporting their involvement in arthritic and rheumatic diseases. TSP-1 is a secreted matricellular protein, and is a member of a family of non-structural ECM proteins. It relays various signals through binding to cell surface receptors or structural matrix proteins [15]. In addition, VDAC1 was selected, although it was identified only in previous research conducted by our group [9] and was not present in the current MS data. VDAC1 is of special interest; it is a member of the same protein family as VDAC2 and plays a role in chondrocyte volume control and in the regulation of apoptosis [16, 17]. The aforementioned selected proteins were validated and quantified using western blotting, since MS analysis with the sample size used in this study is a qualitative rather than quantitative method.

**Fig. 5:**
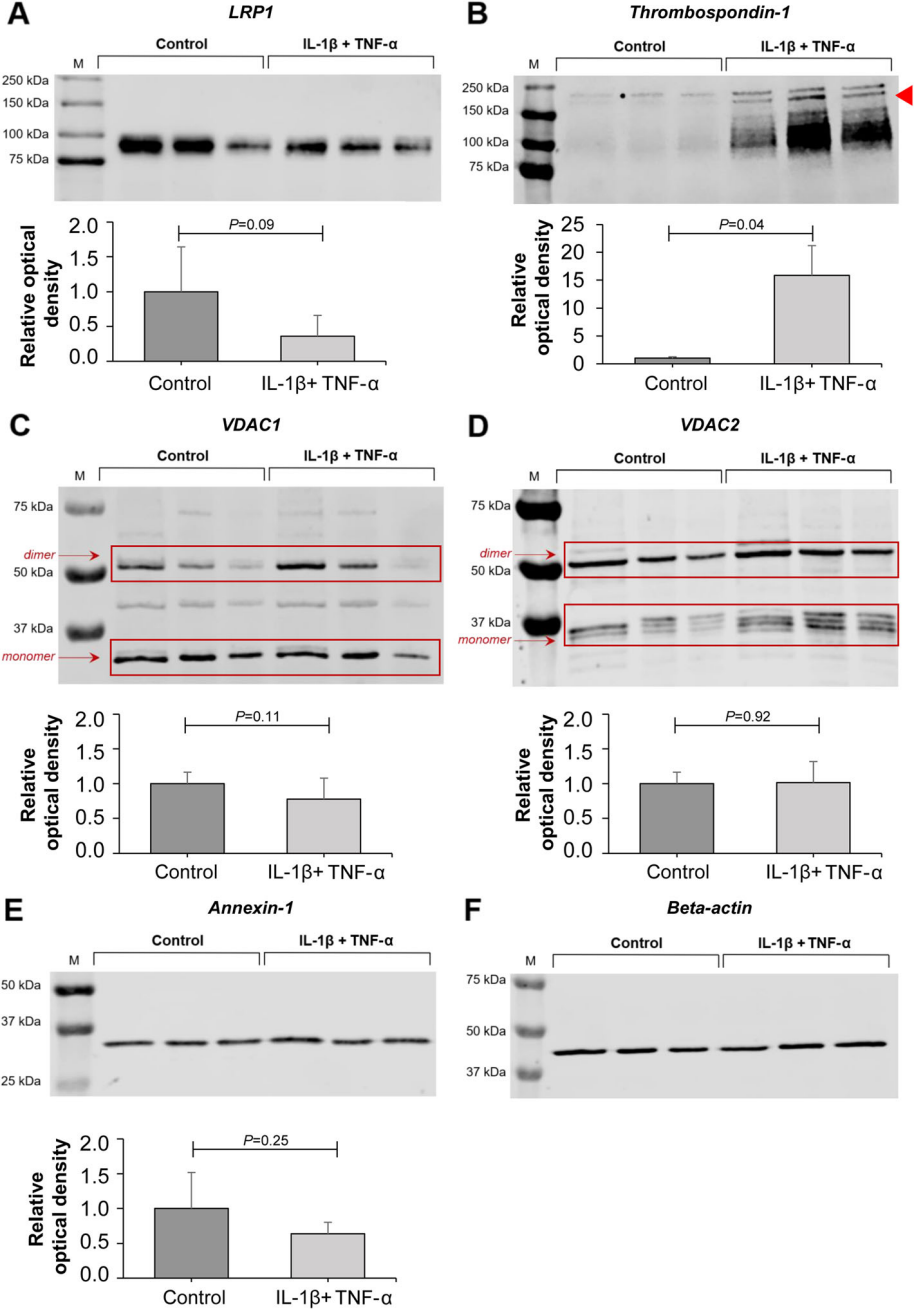
Western blotting and densitometric analysis of LRP-1, thrombospondin, VDAC1, VDAC2 and annexin A1 in cell lysates of chondrocytes under inflammatory (IL-1β + TNF-α, both 10 ng/mL) versus control conditions. **a** Non-significant decrease in LRP-1 expression upon cytokine exposure. **b** Increase in thrombospondin expression upon cytokine exposure. **c** Similar VDAC1 expression. **d** Similar VDAC2 expression. **e** Annexin A1 expression showed a trend to decrease upon cytokine exposure. **f** Beta-actin was measured on each western blot separately as a loading control to correct for the exact amount of protein per lane. Measurements from three horses (three biological replicates) were combined to provide final values for each group (mean ± SD)

**Fig. 6:**
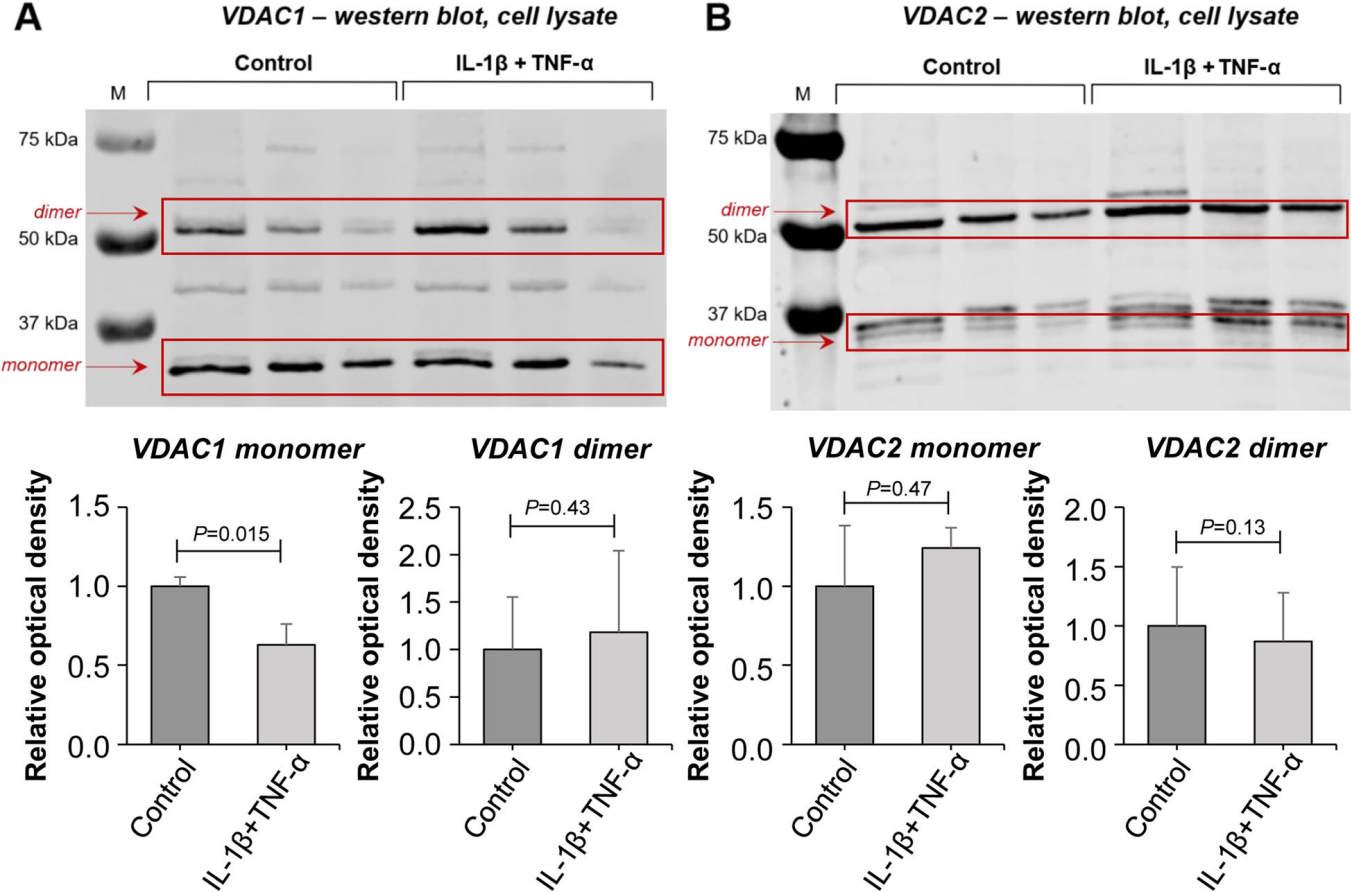
Western blotting and densitometric analysis of VDAC1 and VDAC2 monomers and dimers in cell lysates of chondrocytes upon cytokine exposure (IL-1β + TNF-α, both 10 ng/mL) versus control conditions. **a** VDAC1 monomer expression was significantly decreased upon cytokine exposure. VDAC1 dimer expression was unchanged. **b** Similar VDAC2 monomer and dimer expression. Measurements from three horses (three biological replicates) were combined to provide final values for each group (mean ± SD)

Total cell lysates of pro-inflammatory cytokine-exposed and control chondrocytes for prolow-density lipoprotein receptor-related protein 1 (LRP-1) showed a protein band around 85 kDa on western blots, corresponding to its predicted molecular mass (Fig. 5a). Densitometry showed that LRP-1 level was decreased in response to cytokine exposure. However, this difference did not reach statistical significance, due to the large standard deviation in control chondrocytes (*P* = 0.09). Western blotting of TSP-1 (155 kDa protein band) indicated a clear increase in its levels in cytokine-exposed chondrocytes (*P* = 0.04) (Fig. 5b, arrowhead). VDAC1 expression (predicted molecular mass: 31 kDa protein band) did not differ between the lysates of cytokine-exposed and control chondrocytes (*p* = 0.11) (Fig. 5c). However, when monomers (31 kDa band) and dimers [18] (~ 60 kDa protein band) were analysed separately, monomer levels were decreased by 37% in response to cytokine treatment (*P* = 0.015), while dimer levels did not change (*P* = 0.43) (Fig. 6a). Of note, VDAC1 levels in sample 3 (both control and cytokine-treated) were lower compared to samples 1 and 2, which is probably a result of an individual variation. VDAC2 expression (predicted molecular mass: 31 kDa protein band) did not differ between the lysate of cytokine-treated and control chondrocytes (*P* = 0.92) (Fig. 5d). Analysis of monomer and dimer isoforms [19] (~ 55 kDa protein band) separately also showed no significant difference in protein levels (*P* = 0.47 and *P* = 0.13, respectively) (Fig. 6b). Annexin A1 (35 kDa protein band) levels were also unchanged in response to cytokine treatment (*P* = 0.25) (Fig. 5e).

## Discussion

According to the results presented above, articular chondrocytes exposed to the pro-inflammatory mediators interleukin-1-beta (IL-1β) and tumour necrosis factor-alpha (TNF-α) are characterized by a different assembly of cell surface ion channels and transporters. A high number of proteins exhibited altered expression under inflammatory conditions.

This is the first study that has characterized the surfaceome of primary equine articular chondrocytes upon stimulation with pro-inflammatory cytokine as compared to control conditions. Our results provide the first repository of proteomic data on differentially expressed low-abundance proteins, such as receptors, ion channels and transporter molecules on the surface of chondrocytes in response to pro-inflammatory stimuli. Applying a state-of-the art membrane protein isolation technique using AOB labelling followed by LC/MS-MS proteomics, resulted in improved enrichment of cell surface proteins (60% of identified proteins) as compared to previous methods [9]. The proportion of surfaceome proteins was comparable to our previous study, which describes the complement of cell surface proteins enriched using the same methodology on stem cells [10]. Up to 431 unique surface proteins were reliably identified, including low-abundance proteins, such as ion channel subunits, receptors and transporter molecules. A substantial number of the cell surface proteins identified showed differential expression pattern following cytokine exposure. This is in agreement with published data suggesting that several genes encoding ion channels that are involved in the regulation of mechanotransduction, cell volume, resting membrane potential (RMP) and apoptosis are differentially expressed in OA chondrocytes [20]. In particular, the activation of a volume-sensitive Cl^−^ conductance was reported in a rabbit OA model prior to the onset of macroscopic OA [21]. Despite these data, our current understanding of the surfaceome of primary articular chondrocytes, especially with regards to differentially regulated ion channels, receptors and transporters in OA, is currently lacking.

As in our previous studies [22], treatment with IL-1β and TNF-α was used to mimic the pro-inflammatory micro-environment of cartilage in OA. These two pro-inflammatory cytokines have been shown to be produced by chondrocytes in vivo and in vitro [23] and play a central role in OA pathology [24, 25]. One of their key roles in OA is the induction of MMPs and aggrecanases [26–28] via protein kinase C (PKC) mediated activation of NF-κB and MAPK pathways [29]. Increased levels of key MMPs, such as MMP-1, MMP-3 and MMP-13, lead to intensified proteolysis and progressive loss of cartilage ECM [30, 31], one of the hallmarks of OA.

Validation of the inflammatory micro-environment used in this study and the phenotypic changes induced in chondrocytes exposed to pro-inflammatory cytokines was achieved by detecting and quantifying MMP-1, MMP-3, and MMP-13 in the secretome of chondrocytes under both pro-inflammatory and control conditions. Whilst levels of both MMP-3 and MMP-13 were significantly elevated after cytokine treatment, total MMP-1 showed no change. However, the ‘superactive’ form of MMP-1, which is an activated form of the proenzyme followed by enzymatic cleavage and stabilised by a salt bridge [11, 12], was present only under inflammatory conditions, most probably indicating an increase in MMP-1 activity. The model was additionally validated by showing that GAG production and release into the chondrocyte secretome decreases under pro-inflammatory conditions compared to the control. These findings are in line with other studies demonstrating that inflammatory conditions lead to a reduction in new GAG synthesis by chondrocytes [32], in addition to the loss of major GAG-bearing regions of aggrecan [33, 34].

Pathway over-representation analysis using the Reactome resource also provided an additional layer of validation; one pathway which was enriched in the cytokine-treated chondrocytes was ‘ATF6-alpha activates chaperones.’ ATF6 has been described as a mediator in endoplasmic reticulum (ER) stress-mediated apoptosis in osteoarthritic cartilage via the transcription factor X-box binding protein-1 (XBP1S) [35]. Another over-represented pathway in chondrocytes under inflammatory conditions was ‘Regulation of Insulin-like Growth Factor (IGF) transport and uptake by Insulin-like Growth Factor Binding Proteins (IGFBPs)’. Indeed, increased IGFBP levels have been reported in the articular cartilage and synovial fluid from patients with OA, contributing to the degenerative changes in OA cartilage [36].

The equine articular chondrocyte surfaceome proteins identified in this study both confirms earlier findings and, when compared to our previous work, adds a number of novel PM proteins to the cell surface subproteome arsenal, including zinc transporter ZIP14 (S39AE), cationic amino acid transporter CAT-2 (CTR2), anion exchange protein AE-2 (B3A2), CD109, and disintegrin and metalloproteinase domain-containing protein 17 (ADAM-17) [9]. Additionally, for the first time, it was possible to demonstrate differences in cell surface protein expression between chondrocytes upon pro-inflammatory cytokine exposure as compared to control conditions. Given that the LC-MS/MS approach with the sample size used in the current study did not enable bona fide quantitative comparison between control and pro-inflammatory cytokine-treated samples, we validated a selection of cell surface proteins using western blot analysis, including TSP-1, LRP-1, annexin A1, VDAC1 and VDAC2.

Thrombospondin-1 (TSP-1) is a multifunctional matricellular protein mediating various cell-to-cell and cell-to-matrix interactions [37, 38], but is also believed to have functional roles in cell migration [39], differentiation, proliferation and cell death via the transforming growth factor beta (TGF-β) and other pathways [37, 40, 41]. Adenovirus-mediated intraarticular gene transfer of TSP-1 significantly suppressed OA progression in a rat model of OA [42]. TSP-1 levels have previously been shown to be increased in the secretome of equine articular chondrocytes under inflammatory conditions [43] as well as in osteoarthritic human cartilage samples when compared to the control [44]. Our western blot results confirmed these findings. Furthermore, TSP-1 can bind to the cell surface via LRP-1, a member of the low-density lipoprotein receptor (LDLR) family [45]. LRP-1 internalizes and rapidly degrades TSP-1. Intriguingly, our data also shows a marked decrease in LRP-1, possibly responsible for the decrease in TSP-1 degradation.

LRP-1 is also an interesting PM protein because it can be partially secreted as a soluble fragment. It mediates internalization of many other proteins, including proteinases, lipoproteins, ECM proteins and cell surface receptors [46]. By internalizing and degrading proteins such as ADAMTS-4, ADAMTS-5, MMP-13 and TIMP3, LRP-1 acts as a key mediator of cartilage turnover [47–49]. Furthermore, LRP-1 also controls the canonical Wnt/β-catenin signalling pathway by interacting with Frizzled-1 [50] and connective tissue growth factor [51], both of which regulate endochondral ossification and articular cartilage regeneration, making them vital in skeletal development and in the maintenance of cartilage homeostasis. Messenger RNA levels of LRP-1 have been found to be unaltered in normal and OA human cartilage, while levels of LRP-1 protein were reduced in OA cartilage [52]. Our western blot data also revealed a decrease in LRP-1, although the difference was nominal. This decrease could have been caused by ectodomain shedding of LRP-1 from the cell surface by regulated proteolysis, a phenomenon that often occurs in order to regulate a variety of cellular and physiological functions [53] and could have potential for further development as a biomarker.

Other identified and validated PM proteins include annexin A1, VDAC1 and VDAC2. However, these cell surface proteins might be less important from the perspective of developing a diagnostic and/or prognostic biomarker, since they are not known to be secreted as soluble fragments. Yet, they could be still valuable pharmaceutical targets. Annexin A1 is a member of the annexin superfamily of Ca^2+^-dependent phospholipid binding proteins. It was discovered as a second messenger to facilitate the anti-inflammatory effect of glucocorticoids [54, 55]. In addition, it is a key inhibitory regulator of the immune system [56–58]. Furthermore, annexin-1 is involved in exocytosis, inflammatory signalling, cell proliferation and apoptosis [59–61], emphasizing the multifunctional nature of this protein [59]. It has been found that annexin A1 is present at lower abundance in human OA cartilage as compared to control cartilage [62]. Our western blot results showed a trend towards a decrease in annexin-1 expression, although this was not statistically significant.

VDAC1 was identified as a chondrocyte PM protein in previous research conducted by our group [9, 63], and it was also detected in the surfaceome of mesenchymal stem cells [10]. VDAC1 is the dominant member of the VDAC family, consisting of VDAC1–3. It is a pore-forming protein, which was originally discovered in the outer mitochondrial membrane (OMM) [64]. Here, it is involved in the transport of anions, cations, ATP, Ca^2+^ and metabolites, depending on its configuration (open/closed), conductance, and interactions [65]. As such, VDAC1 regulates mitochondrial function and contributes importantly to the metabolic phenotype of the cell [18]. Furthermore, VDAC1 is a key protein in mitochondria-mediated apoptosis, although findings are controversial and mechanisms remain poorly understood [66]. However, increased Ca^2+^ uptake via VDAC1 across the OMM [67], as well as both VDAC1-mediated reactive oxygen species (ROS) release [68] and pro-apoptotic Bcl-2 family protein activation, has been shown to induce cytochrome C release, leading to caspase activation and ultimately apoptosis. Since cytochrome C is too large to pass through monomeric VDAC1, oligomerization of VDAC1 has been thought to occur in response to specific (apoptotic) signals, such as TNF-α and H_2_O_2_ [69, 70]. A reduced monomeric VDAC1 expression in cytokine-treated chondrocytes was shown by our western blot results. This suggests an increase in oligomerization, although we did not find an increase in VDAC1 dimers. Zalk et al. showed that VDACs also exist in tetramers, so the increase in total VDAC1 expression might mainly represent an increase in tetrameric VDAC1 levels [71]. According to the current UniProt database entries VDACs are localised in the OMM and thence they were not considered as constituents of the surfaceome in this study (and are therefore not listed in Tables S1–S6 in Additional file 1). However, accumulating evidence suggests that VDACs are “moonlighting proteins” [72, 73], and are also located in the PM [9, 18, 74–76]. These proteins are thought to play roles in cell volume regulation by influencing ATP release in response to mechanical stimuli [17]. Additionally, PM-located VDAC1 shows NADH:ferricyanide reductase activity involved in regulating apoptosis [16, 64]. Yet, the potential role of PM-located VDACs in apoptosis remains highly controversial [74, 77].

As far as VDAC2 is concerned, no alterations in pro-inflammatory cytokine-exposed chondrocytes were found when total cell lysates were analysed in western blotting. Very little is known about the function of PM-specific VDAC2 [74, 78]. In our in vitro model, a trend towards increased apoptotic rate was found in cytokine-treated chondrocytes (see Figure S2 in the Supplementary Material, Additional file 1), which is in agreement with previous research [79]. Therefore, it could be hypothesized that PM-specific VDAC2 also plays a role in apoptosis, like PM-specific VDAC1. The focus of future mechanistic studies will be to unravel the role of VDAC2 in chondrocyte apoptosis.

## Conclusions

Using AOB labelling followed by high throughput mass spectrometric analysis we reliably identified a high percentage of PM proteins in the primary equine articular chondrocyte surfaceome under control and pro-inflammatory conditions, including low-abundance PM proteins We were able to describe many cell surface proteins previously unconfirmed in chondocytes. These findings were considered reliable, as we were also able to confirm the presence of those PM proteins (e.g. VDAC1–2) using western blotting that were identified in previous work conducted by our group [9, 10]. However, there are also some limitations to our approach. A number of non-PM proteins were also identified, despite the targeted approach. The most plausible explanation for this is the high sensitivity of MS to detect exposed proteins due to cell lysis or death, or the non-PM proteins are interacting partners for the proteins constituting the chondrocyte surfaceome. Whilst this has also been shown for other cell surface proteome approaches such as cell surface shaving, but the biotinylation method was the most effective in extracting surface proteins [80, 81]. Another possible disadvantage is the use of equine articular chondrocytes. There might be subtle differences between species, potentially limiting translation of our findings to human conditions. Conversely, it has been shown that equine and human cartilage structure and maturation are highly similar [82].

In conclusion, our data add substantially to the elaborate and expanding chondrocyte proteome and enrich the publicly accessible proteomic databases. Above all, this is the first study that investigates chondrocyte surface protein expression upon pro-inflammatory cytokine exposure. Cell surface proteins that were found to be up-regulated or down-regulated can help to develop a better understanding of the pathogenesis of OA and other arthritic diseases. Furthermore, PM proteins that are upregulated and can be secreted, such as LRP-1, might be useful early biomarkers and indicators of catabolic responses associated with OA. Further research will investigate the presence of LRP-1 in human articular cartilage, whether it is measurable extracellularly, and whether it is able to assess disease progression and distinguish between OA and other joint diseases. The proteins that are upregulated may be used as molecular biomarkers of ongoing phenotypic changes in response to an inflammatory micro-environment. Proteins that exhibit reduced expression may be part of repair mechanisms that have been compromised in these conditions. Knowledge of the chondrocyte surfaceome could provide potentially novel targets for future pharmaceutical development or the repositioning of existing drugs. Further validation of identified cell surface proteins is therefore recommended followed by investigation of their functional properties in chondrocytes under inflammatory and control conditions.

## Methods

### Isolation and culture of primary equine articular chondrocytes

Chondrocytes were isolated from macroscopically healthy equine articular cartilage derived from three horses. The animals for this study were euthanized in a UK-based abattoir for purposes other than research. All procedures were carried out in accordance with Welfare of Animals (Slaughter or Killing) Regulations 1995. Ethical approval for the use of abattoir-derived animal tissues was obtained from the Ethics Committee of the School of Veterinary Science and Medicine, University of Surrey. After opening the metacarpophalangeal joint cavity under aseptic conditions, full thickness 8-mm diameter articular cartilage biopsies were taken from the distal end of the metacarpal bone using a sterile biopsy punch and placed in serum-free Dulbecco’s Modified Eagle Medium (DMEM, Thermo Fisher Scientific, Waltham, MA, USA) supplemented with 5% penicillin/streptomycin solution (P/S, Sigma-Aldrich, St. Louis, MO, USA) and 0.05% gentamycin (Sigma-Aldrich) pre-warmed to 37 °C as described previously [22]. Cartilage shavings were washed three times with DMEM containing 5% P/S and 0.05% gentamycin. Chondrocytes were isolated by 1 hour incubation at 37 °C with pronase (from *Streptomyces griseus*; Roche, Basel, Switzerland), followed by overnight incubation at 37 °C with 0.3% type II collagenase (from *Clostridium histolyticum*; Invitrogen, Carlsbad, CA, USA), both dissolved in serum-free DMEM solution containing 1% P/S and 0.05% gentamycin. Following dissociation of cartilage shavings by trituration, the solution was filtered through a 70-μm nylon mesh filter to yield a single-cell suspension, and centrifuged at 2000×*g* for 5 min at room temperature. After washing in serum-free DMEM twice, cells were resuspended in 4.5 g/L glucose DMEM containing 10% foetal calf serum (FCS; Invitrogen) and 1% P/S solution, seeded into tissue culture flasks (Nunc; Thermo Fisher Scientific), and cultured in a 5% CO_2_ incubator at 37 °C. Cells were subcultured when they reached approximately 80% confluence. The medium was changed at least twice a week during cell expansion and passage. Cells from the second passage were used for the experiments. Chondrocytes in the experimental group were treated with IL-1β and TNF-α (both at 10 ng/mL) (equine recombinant, R&D Systems, Minneapolis, MN, USA) for the duration of either 72 h (for membrane protein labelling) or 7 days (for validation of selected proteins by western blotting).

### Validation of catabolic protein markers in chondrocyte monolayer cultures exposed to pro-inflammatory cytokines using western blots and DMMB assays

To confirm that IL-1β and TNF-α induce an inflammatory phenotype in chondrocytes, the expression of the catabolic enzymes MMP-1, MMP-3, and MMP-13 was analysed by western blotting using the culture medium (the “secretome”) of second passage primary articular chondrocytes derived from three horses and used as three biological replicates. Second passage chondrocytes were treated with IL-1β and TNF-α (both at 10 ng/mL) for 7 days to allow the accumulation of secreted MMPs in the culture medium to be detectable with western blotting. Conditioned culture medium was collected, an MMP inhibitor (1:100; Roche) and Complete Protease Inhibitor Cocktail (1:100; Roche) were added, and medium was stored at − 80 °C until analysis.

For each secretome sample, 6× Laemmli sample buffer (375 mM Tris-HCl, pH 6.8, 9% SDS, 50% glycerol, 0.03% bromophenol blue) was added. Subsequently, 0.15 M dithiothreitol (DTT) was added, followed by heating for 5 min at 95 °C. Twenty micrograms of protein for each sample was loaded into a 10-well 10% SDS–PAGE gel for immunological detection of selected proteins. Proteins were transferred to nitrocellulose membranes (Bio-Rad). Membranes were blocked in Odyssey™ blocking buffer (LI-COR Biosciences, Lincoln, NE, USA) in TBST, followed by incubation with the primary antibody in blocking solution at 4 °C overnight, with gentle rotation (for the antibodies used in this study, see Table 1). Membranes were then washed and incubated with the secondary antibody in blocking solution at room temperature for 1 hour (see Table 1). Membranes were rinsed four times followed by three 5-min washes in TBST 0.1% and imaged and quantified using the LI-COR Odyssey™ FC fluorescent imaging system. Beta-actin was measured in the cell lysate from each sample (see below) to control for cell number as a housekeeping protein is not available for secreted molecules. Measurements from three horses (three replicates) were combined to provide final values for each group.

**Table 1:** Detailed specification of primary and secondary antibodies employed for western blotting

Primary antibody	Supplier	Catalog number	Dilution	Secondary antibody	Supplier	Catalog number	Dilution
Annexin A1	LifeSpan Biosciences	LS-C382041 (polyclonal)	1:500	IRDye 800CW Goat	LI-COR Biosciences	926–32,211	1:10,000
				Anti-Rabbit IgG			
LRP1	Abcam	ab92544 (monoclonal)	1:400	IRDye 680RD Goat	LI-COR Biosciences	926–68,070	1:8000
				Anti-Mouse IgG			
MMP-1 (C-terminal)	Aviva Systems Biology	ARP42040 (polyclonal)	1:1000	IRDye 800CW Goat	LI-COR Biosciences	926–32,211	1:10,000
				Anti-Rabbit IgG			
MMP-13 (middle region)	Aviva Systems Biology	ARP56350 (polyclonal)	1:500	IRDye 800CW Goat	LI-COR Biosciences	926–32,211	1:10,000
				Anti-Rabbit IgG			
MMP-3 (middle region)	Aviva Systems Biology	ARP42042 (polyclonal)	1:1000	IRDye 800CW Goat	LI-COR Biosciences	926–32,211	1:10,000
				Anti-Rabbit IgG			
TSP-1	Abcam	ab1823 (monoclonal)	1:500^a^;	IRDye 680RD Goat	LI-COR Biosciences	926–68,070^a^;	1:5000^a^
			1:1000^b^	Anti-Mouse IgG^a^;			
				IRDye 800CW Goat		926–32,210^b^	1:10,000^b^
				Anti-Mouse IgG^b^			
VDAC1 (C-terminal)	Aviva Systems Biology	ARP35122 (polyclonal)	1:250	IRDye 680RD Goat	LI-COR Biosciences	926–68,071	1:5000
				Anti-Rabbit IgG			
VDAC2 (N-terminal)	Aviva Systems Biology	ARP35123 (polyclonal)	1:250	IRDye 680RD Goat	LI-COR Biosciences	926–68,071	1:5000
				Anti-Rabbit IgG			
β-actin	LI-COR Biosciences	926–42,210 (monoclonal)	1:500	IRDye 800CW Goat	LI-COR Biosciences	926–32,211	1:10,000
				Anti-Rabbit IgG			

^a^Employed on total cell lysates. ^b^Employed on conditioned media samples

Furthermore, to evaluate the release of proteoglycans in the chondrocyte secretome, the sulphated glycosaminoglycan (sGAG) concentration was quantified using the metachromatic dye 1,9-dimethyl-methylene blue (DMMB) assay (Sigma-Aldrich). DMMB and shark chondroitin sulphate standards were prepared as described previously [83]. Chondrocyte media samples were diluted to appropriate concentrations, to be within the accurate range (0 to 40 μg/mL) of the standard curve. On a 96-well plate, 20 μL of each standard (in triplicates) and sample dilutions (also in triplicates) were added, followed by 200 μL of DMMB solution. The absorption of the samples was read at 525 nm within 10 min using a Tecan SPARK 10 M Plate Reader (Tecan, Männedorf, Switzerland). Measurements from three horses (four replicates per treatment group) were combined to provide final values for each group.

### Sample preparation, AOB labelling of membrane proteins and trypsin digestion of proteins for LC-MS/MS analysis

Second passage primary equine articular chondrocytes were cultured in T175 flasks for 72 h with or without IL-1β and TNF-α (both at 10 ng/mL), until approximately 80% confluence. Cultures were washed twice with PBS and then incubated in serum-free medium with or without IL1-β and TNF-α (both at 10 ng/mL) for 1 h at 37 °C. The samples for western blotting were paired: for each pair, controls and cytokine-treated samples were obtained from the same horse for each biological replicate. Unless indicated otherwise, all reagents below were obtained from Thermo Fisher Scientific. After washing cells twice with ice-cold PBS, 4 mL oxidation/biotinylation mix was added containing 1 mM sodium meta-periodate, 100 mM AOB and 10 mM aniline in ice-cold PBS. Cells were incubated, rocking in the dark at 4 °C for 1 h to oxidize and biotinylate surface sialic acid residues. The oxidation reaction was quenched by the addition of glycerol to a final concentration of 1 mM and incubated for 10 min. Cells were washed with PBS and then with PBS containing 1 mM CaCl_2_ and 0.5 mM MgCl_2_. Cells were scraped off the flask and collected by centrifuging at 300×*g* at room temperature for 7 min. The resulting cell pellet was resuspended in lysis buffer (1% Triton X-100, 150 mM NaCl, 1× protease inhibitor, 5 mM iodoacetamide, 0.1 mg/mL PMSF and 10 mM Tris-HCl pH 7.6), and incubated at 4 °C for 30 min. Adequate lysis was achieved by vortexing/pipetting the cell pellet in lysis buffer every 5 min. Cell debris and nuclei were removed by centrifugation at 4 °C once at 2800×*g* for 10 min and once at 16,000×*g* for 10 min.

To isolate labelled glycoproteins, 300 μL of NeutrAvidin agarose beads were added to Snap Cap spin columns followed by three washes with PBS and incubation with the cell lysate for 2 h at 4 °C. Non-specifically bound proteins were eliminated by multiple washing steps each time followed by centrifugation at 1000×*g* for 30 s. Washing steps included consecutively 10× lysis buffer, 10× PBS/0.5% SDS followed by a 20 min incubation at room temperature with PBS/0.5% SDS/100 mM DTT, 10× UC buffer (consisting of 6 M urea, 100 mM Tris-HCl; pH 8.5), followed by alkylation for 20 min at room temperature with UC buffer containing 50 mM iodoacetamide, 10× UC buffer, 10× 5 M NaCl, 100 mM Na_2_CO_3_, 10× PBS and 10× HPLC grade water. Biotinylated glycoproteins were digested on-beads overnight at 37 °C in 50 mM NH_4_HCO_3_ containing 5 μg Trypsin Gold (Promega, Madison, WI, USA). Tryptic peptides were collected via centrifugation at 1000×*g* for 1 min at 4 °C. Beads were rinsed with 50 mM NH_4_HCO_3_ and tryptic fractions pooled. Clean up of peptide samples was performed using C18 spin columns according to the manufacturer’s protocol. Samples were dried gently in a vacuum evaporator and subsequently stored at − 80 °C until analysis. The surface protein enrichment protocol was performed on cells obtained from two different horses (2 biological replicates).

### LC-MS/MS analysis

Samples were injected into a 15 cm C18 Pepmap column using a Dionex UltiMate® 3000 RSLCnano chromatography platform with a flow rate of 300 nL/min to separate peptides. Three microliter of each sample was injected into the HPLC column. After peptide binding and washing on the column, the complex peptide mixture was separated and eluted by a gradient of solution A (100% water + 0.1% formic acid) and solution B (100% acetonitrile + 0.1% formic acid) over 115 min, followed by column washing and re-equilibration. The peptides were delivered to a Bruker amaZon ETD ion trap instrument. The top 5 most intense ions from each MS scan were selected for fragmentation. The nanoLC-MS/MS analysis was performed on two biological replicate samples.

### Peptide and protein identification, data analysis and bioinformatics

Processed data from the analysed samples were compiled into MGF files, which were converted to the mzML format using MSConvert (ProteoWizard 3.0.18212), and then filtered, de novo sequenced and assigned with protein ID using PEAKS Studio 8.5 software [84] (Bioinformatics Solutions, Waterloo, Canada), by searching against the mammalian SwissProt database (March 2018; 20,314 entries), with the parameters of fixed modification carbamidomethylation of cysteine and variable modifications of methionine oxidation and deamidation (NQ). This result was then processed with PEAKS PTM which looks at all 313 naturally occurring modifications in Unimod. The parent mass tolerance was set to 15 ppm using monoisotopic mass, and a fragment ion mass tolerance was set to 0.1 Da. Data were validated using the FDR method built in PEAKS 8.5 in which protein identifications were accepted with a confidence score (−10lgP) > 20 for peptides and (−10lgP) > 15 for proteins, with at least 1 peptide per protein.

Given that the horse genome has not yet been fully sequenced and consequently the proteomic database is also incomplete, we undertook cross-species protein identification to capture a higher number of protein IDs. This approach is acceptable as the level of similarity between closely related species is high (e.g., mouse-human comparisons reveal approx. 70% identity) [85, 86]. To this end, we have chosen to search the mammalian UniProt database and accepted proteins from the following species (in addition to *Equus caballus*): *Antilope cervicapra, Bos taurus*, *Camelus dromedaries, Canis lupus familiaris, Gorilla gorilla gorilla, Homo sapiens, Macaca fascicularis, Mesocricetus auratus*, *Mus musculus, Oryctolagus cuniculus, Rattus norvegicus,* and *Sus scrofa.* After MS identification and database searches, 1819 unique proteins were identified in the 4 samples combined. Proteins that were positively identified in at least one sample but were assigned to more than one species were counted as a single protein ID. Since the aim of applying a discovery-based shotgun proteomics approach in this study was to generate a list of the overall proteins expressed on the surface of equine chondrocytes, and given the very low abundance of certain surface proteins, we included every protein into the lists that have been reliably identified in at least one biological replicate. If a protein was present above the limit of detection in a sample, given that trypsin has been used for on-bead digestion, the probability of at least one peptide being selected for fragmentation is high.

Seven hundred twenty-three unique proteins were manually classified per cellular compartment and/or function using the UniProt (http://www.uniprot.org) database and gene ontology (GO) annotations, considering homologous proteins and literature data. GO terms, describing molecular function and subcellular localisation, were downloaded from UniProt. Some proteins were difficult to classify into a single cellular compartment and/or function, because accurate predictions were limited and experimental evidence lacking. Also, many proteins may be present in multiple cellular compartments. When proteins were found to be present in the PM, this localisation was considered the most likely, since selective surfaceome protein extraction was attempted. GO molecular function data entries were used to categorise the proteins into three functional classes (receptors, enzymes, and transporters), one extracellular matrix component class, and one structural/adhesion protein class, based on already published classification criteria [87]. Proteins that did not fit into any of the above functional classes were marked as unclassified. The following key words were used to assign proteins into the above categories: for *transporters*, the key words ‘transporter,’ ‘symporter,’ ‘antiporter,’ ‘channel,’ ‘porin,’ and ‘exchanging’ were used; for *receptors*, the key word ‘receptor’ was used; for *enzymes*, we searched for the key word ‘enzymatic activity’; for ECM components, the key words ‘extracellular space,’ and/or ‘extracellular matrix’ were applied; and for the group of Structural/Adhesion/Junctional proteins ‘cell-cell junction,’ ‘adherens junction,’ ‘focal adhesion’ and ‘cytoskeleton’ were used.

Given that the GO annotations for certain proteins contained multiple entries, for some proteins we were unable to assign a single molecular function. This resulted in some proteins being included in more than one list. For example, EPHA2, the ephrin type-A receptor 2 entry is listed under both enzymes and receptors as ephrin type-A receptors belong to the class of receptor tyrosine kinases.

The mass spectrometry proteomics data have been deposited to the ProteomeXchange Consortium via the PRIDE [88] partner repository with the dataset identifier PXD014773.

For pathway over-representation analysis, we entered the curated data to the Reactome resource (https://reactome.org/). We first used UniProt to map the mammalian protein accession numbers to their genes. These lists were submitted for pathway over-representation analyses. The 25 most relevant pathways sorted by *p*-value for both control and cytokine-treated conditions are shown in Tables 2 and 3, and over-represented pathways are also shown in a foam tree format in Additional files 2 and 3. In addition, we also submitted the surfaceome data to STRING (https://string-db.org/) to check for protein association networks. Given that the Uniprot database was originally searched against Mammalia (since the *Equus* genome and proteome has not been fully resolved), and the protein IDs were from multiple species, we first converted the proteins to their human equivalent based on closest homology. Then, we submitted this list of IDs to STRING to predict protein-protein interactions. We increased the confidence setting from default (4.00) to high (9.00) to try to pull out the main interactors. The analyses for control and cytokine-treated conditions (unique proteins) are shown in Additional files 4 and 5. The UniProt-converted protein lists to their human gene equivalents are available in Additional file 6.

**Table 2:** Results of an overrepresentation analysis on the identified proteins in unstimulated equine articular chondrocytes carried out using the Reactome resource. The analysis is based on a statistical (hypergeometric distribution) test that determines whether certain Reactome pathways are over-represented (enriched) in the submitted data. This test produces a probability score, which is corrected for false discovery rate using the Benjamani-Hochberg method. The 25 most relevant pathways sorted by *p*-value are shown. Table extracted from Reactome Pathway Analysis Report

Pathway name	Entities				Reactions	
	found	ratio	*p*-value	FDR^a^	found	ratio
Neutrophil degranulation	66 / 480	0.033	1.11e-16	1.51e-13	10 / 10	8.01e-04
Formation of the cornified envelope	33 / 138	0.01	7.77e-16	5.29e-13	11 / 27	0.002
Extracellular matrix organization	47 / 329	0.023	1.37e-13	6.24e-11	181 / 318	0.025
Integrin cell surface interactions	21 / 86	0.006	1.02e-10	3.47e-08	46 / 54	0.004
Axon guidance	59 / 584	0.04	1.33e-10	3.61e-08	182 / 297	0.024
Keratinization	33 / 226	0.016	3.80e-10	8.64e-08	18 / 34	0.003
Developmental Biology	92 / 1207	0.084	2.00e-09	3.88e-07	206 / 511	0.041
Platelet degranulation	24 / 137	0.009	3.30e-09	5.61e-07	8 / 11	8.81e-04
Response to elevated platelet cytosolic Ca2+	24 / 144	0.01	8.45e-09	1.28e-06	8 / 14	0.001
Platelet activation, signaling and aggregation	35 / 293	0.02	1.76e-08	2.40e-06	42 / 114	0.009
Interleukin-12 family signaling	18 / 96	0.007	1.13e-07	1.39e-05	33 / 114	0.009
EPH-Ephrin signaling	18 / 101	0.007	2.35e-07	2.66e-05	52 / 56	0.004
Innate Immune System	91 / 1328	0.092	3.22e-07	3.35e-05	174 / 696	0.056
Cell junction organization	17 / 94	0.007	4.15e-07	4.02e-05	26 / 37	0.003
Signaling by Receptor Tyrosine Kinases	48 / 554	0.038	6.84e-07	6.03e-05	261 / 657	0.053
Cell-Cell communication	20 / 133	0.009	7.12e-07	6.03e-05	36 / 60	0.005
Hemostasis	63 / 821	0.057	7.54e-07	6.03e-05	83 / 327	0.026
Non-integrin membrane-ECM interactions	13 / 61	0.004	1.64e-06	1.23e-04	16 / 22	0.002
Gene and protein expression by JAK-STAT signaling after Interleukin-12 stimulation	14 / 73	0.005	2.21e-06	1.56e-04	7 / 36	0.003
Interleukin-12 signaling	15 / 84	0.006	2.29e-06	1.56e-04	9 / 56	0.004
EPH-ephrin mediated repulsion of cells	12 / 55	0.004	3.23e-06	2.06e-04	9 / 9	7.21e-04
Syndecan interactions	9 / 29	0.002	3.47e-06	2.06e-04	9 / 15	0.001
Degradation of the extracellular matrix	20 / 148	0.01	3.49e-06	2.06e-04	53 / 105	0.008
Immune System	156 / 2822	0.196	7.43e-06	4.16e-04	344 / 1597	0.128
L1CAM interactions	18 / 130	0.009	7.70e-06	4.16e-04	21 / 54	0.004

^a^False Discovery Rate

**Table 3:** Results of an overrepresentation analysis on the identified proteins following cytokine stimulation in equine articular chondrocytes carried out using the Reactome resource. The analysis is based on a statistical (hypergeometric distribution) test that determines whether certain Reactome pathways are over-represented (enriched) in the submitted data. This test produces a probability score, which is corrected for false discovery rate using the Benjamani-Hochberg method. The 25 most relevant pathways sorted by *p*-value are shown. Table extracted from Reactome Pathway Analysis Report

Pathway name	Entities				Reactions	
	found	ratio	*p*-value	FDR^a^	found	ratio
Neutrophil degranulation	56 / 480	0.033	1.11e-16	1.27e-13	10 / 10	8.01e-04
Formation of the cornified envelope	28 / 138	0.01	1.94e-14	1.11e-11	13 / 27	0.002
Extracellular matrix organization	42 / 329	0.023	4.09e-14	1.56e-11	183 / 318	0.025
RAB geranylgeranylation	18 / 68	0.005	1.39e-11	3.99e-09	2 / 5	4.00e-04
Integrin cell surface interactions	18 / 86	0.006	5.71e-10	1.31e-07	45 / 54	0.004
Keratinization	28 / 226	0.016	1.55e-09	2.97e-07	20 / 34	0.003
Non-integrin membrane-ECM interactions	15 / 61	0.004	1.88e-09	3.06e-07	19 / 22	0.002
Platelet degranulation	21 / 137	0.009	5.09e-09	7.28e-07	8 / 11	8.81e-04
Response to elevated platelet cytosolic Ca2+	21 / 144	0.01	1.19e-08	1.51e-06	8 / 14	0.001
Cell-Cell communication	20 / 133	0.009	1.61e-08	1.84e-06	46 / 60	0.005
Syndecan interactions	10 / 29	0.002	4.43e-08	4.61e-06	12 / 15	0.001
Axon guidance	45 / 584	0.04	4.92e-08	4.67e-06	147 / 297	0.024
Developmental Biology	73 / 1207	0.084	6.41e-08	5.64e-06	167 / 511	0.041
Post-translational protein phosphorylation	17 / 109	0.008	1.16e-07	9.37e-06	1 / 1	8.01e-05
Cell junction organization	15 / 94	0.007	4.83e-07	3.67e-05	26 / 37	0.003
Regulation of Insulin-like Growth Factor (IGF) transport and uptake by Insulin-like Growth Factor Binding Proteins (IGFBPs)	17 / 127	0.009	9.31e-07	6.61e-05	1 / 14	0.001
Transport of small molecules	58 / 963	0.067	1.97e-06	1.32e-04	97 / 438	0.035
Insulin-like Growth Factor-2 mRNA Binding Proteins (IGF2BPs/IMPs/VICKZs) bind RNA	6 / 13	9.01e-04	4.45e-06	2.80e-04	2 / 3	2.40e-04
ATF6 (ATF6-alpha) activates chaperone genes	6 / 15	0.001	9.96e-06	5.98e-04	3 / 5	4.00e-04
Vesicle-mediated transport	49 / 824	0.057	1.83e-05	0.001	115 / 251	0.02
Adherens junctions interactions	8 / 35	0.002	1.93e-05	0.001	16 / 16	0.001
ATF6 (ATF6-alpha) activates chaperones	6 / 17	0.001	2.00e-05	0.001	4 / 10	8.01e-04
Transport of inorganic cations/anions and amino acids/oligopeptides	17 / 165	0.011	2.71e-05	0.001	23 / 75	0.006
Platelet activation, signaling and aggregation	24 / 293	0.02	2.78e-05	0.001	33 / 114	0.009
Innate Immune System	69 / 1328	0.092	2.80e-05	0.001	88 / 696	0.056

^a^False Discovery Rate

### Total protein sample preparation and validation of selected proteins by western blotting

Proteins were selected for western blot analysis based on their presence in both cytokine-treated and control samples following mass spectrometry, as well as literature supporting their involvement in arthritic and rheumatic diseases, especially OA. Approximately 80% confluent cultures (T175 flasks) of second-passage primary articular chondrocytes derived from three horses were incubated in DMEM with or without IL-1β and TNF-α (both at 10 ng/mL) for 72 h at 37 °C. After treatment, cells were washed twice with PBS, followed by addition of 1 mL lysis buffer (10% glycerol, 2% SDS, 63 mM Tris-HCl; pH 6.8). Cells were scraped off the flasks and 1:100 MMP inhibitor (Roche) and 1:100 Complete Protease Inhibitor Cocktail (Roche) was added. DNA was sheared using a 25G syringe needle (Becton-Dickinson & Co) and removed by centrifuging at 800×*g* for 5 min. Samples were stored at − 80 °C until analysis. Protein concentration in the samples was determined using the Pierce BCA protein assay kit according to the manufacturer’s protocol (Thermo Fisher Scientific). The absorption values of the assayed samples at 562 nm were read using a Tecan SPARK 10 M Microplate Reader. Western blots were performed as described above, with the exception that low density lipoprotein-related protein-1 (LRP-1) was run under non-reducing conditions. Specifications of all primary and secondary antibodies used are described in Table 1. Beta-actin was measured on each blot separately as loading control. Measurements from three horses (three replicates) were combined to provide final values for each treatment group. Uncropped western blot membrane images for all western blots are shown in Additional file 7: Figures S3–S12 online.

### Statistical analysis

GraphPad Prism 7.02 and Microsoft Excel software were used to produce graphic images and perform paired Student *t*-tests. The data was tested for normality using Shapiro-Wilk test. *P* values < 0.05 were considered statistically significant.

## Supplementary information

**Additional file 1: Figure S1.** GAG release levels in the chondrocyte secretome upon cytokine exposure. **Figure S2.** Rate of apoptosis of chondrocytes under pro-inflammatory versus control conditions. **Table S1.** List of proteins classified as Enzymes based on GO annotations. **Table S2.** List of proteins classified as Receptors based on GO annotations. **Table S3.** List of proteins classified as Transporters based on GO annotations. **Table S4.** List of proteins that could not be classified into any of the previous categories based on GO annotations (listed as Unclassified). **Table S5.** List of proteins classified into the category of Structural/Adhesion/Junctional proteins based on GO annotations. **Table S6.** List of proteins classified into the category of Extracellular matrix proteins based on GO annotations.

**Additional file 2.** Foam tree of the over-represented pathways of the surface proteins identified in untreated control chondrocytes generated by the Reactome resource.

**Additional file 3.** Foam tree of the over-represented pathways of the surface proteins identified in chondrocytes exposed to a pro-inflammatory micro-environment generated by the Reactome resource.

**Additional file 4.** Predicted protein interactions in untreated control chondrocytes generated by the String resource.

**Additional file 5.** Predicted protein interactions in chondrocytes exposed to a pro-inflammatory micro-environment generated by the String resource.

**Additional file 6.** Lists of the UniProt-converted proteins (multiple species) to their human gene equivalents.

**Additional file 7.** This file contains the uncropped western blot membrane images presented in Figs. 2, 5 and 6.

## Data Availability

The mass spectrometry proteomics data have been deposited to the ProteomeXchange Consortium via the PRIDE [88] partner repository with the dataset identifier PXD014773.^1^

## Abbreviations

ADAMTS: A disintegrin and metalloproteinase with thrombospondin motifs
AOB: Aminooxy-biotin
DMMB: 1, 9-dimethyl-methylene blue
ECM: Extracellular matrix
ER: Endoplasmic reticulum
GAG: Glycosaminoglycan
GO: Gene ontology
IGF: Insulin-like growth factor
IGFBP: Insulin-like growth factor binding protein
IL-1β: Interleukin-1 beta
LC-MS/MS: Liquid chromatography and tandem mass spectrometry
LDLR: Low density lipoprotein receptor
LRP-1: Low density lipoprotein related protein 1
MMP: Matrix metalloproteinase
NO: Nitric oxide
OA: Osteoarthritis
OMM: Outer mitochondrial membrane
PKC: Protein kinase C
PM: Plasma membrane
RA: Rheumatoid arthritis
RMP: Resting membrane potential
ROS: Reactive oxygen species
TGF-β: Transforming growth factor beta
TNF-α: Tumour necrosis factor alpha
TSP: Thrombospondin
VDAC: Voltage dependent anion channel
XBP1S: X-box binding protein-1, spliced variant
