# Supplementary material for: Alterations in the chondrocyte surfaceome in response to pro-inflammatory cytokines
Source: BMC Mol Cell Biol. 2020 Jun 26;21:47. doi: 10.1186/s12860-020-00288-9 (PMC7318434; doi:10.1186/s12860-020-00288-9)
Supplement: Supplementary file 1 — Additional file 1: Figure S1. GAG release levels in the chondrocyte secretome upon cytokine exposure. Figure S2. Rate of apoptosis of chondrocytes under pro-inflammatory versus control conditions. Table S1. List of proteins classified as Enzymes based on GO annotations. Table S2. List of proteins classified as Receptors based on GO annotations. Table S3. List of proteins classified as Transporters based on GO annotations. Table S4. List of proteins that could not be classified into any of the previous categories based on GO annotations (listed as Unclassified). Table S5. List of proteins classified into the category of Structural/Adhesion/Junctional proteins based on GO annotations. Table S6. List of proteins classified into the category of Extracellular matrix proteins based on GO annotations. [file 12860_2020_288_MOESM1_ESM.pdf]

Supplementary Materials for

Alterations in the chondrocyte surfaceome in response to pro-inflammatory cytokines

Bernadette Jeremiasse, Csaba Matta*, Christopher R. Fellows, David J. Boocock, Julia R. Smith, Susan Liddell, Floris Lafeber, Willem E. van Spil, Ali Mobasheri

*Corresponding author. E-mail: matta.csaba@med.unideb.hu (C.M.)

This PDF file (Additional File #1) includes:

Figure S1. GAG release levels in the chondrocyte secretome upon cytokine exposure

Figure S2. Rate of apoptosis of chondrocytes under pro-inflammatory versus control conditions

Table S1. List of proteins classified as Enzymes based on GO annotations

Table S2. List of proteins classified as Receptors based on GO annotations

Table S3. List of proteins classified as Transporters based on GO annotations

Table S4. List of proteins that could not be classified into any of the previous categories based on GO annotations (listed as Unclassified)

Table S5. List of proteins classified into the category of Structural/Adhesion/Junctional proteins based on GO annotations

Table S6. List of proteins classified into the category of Extracellular matrix proteins based on GO annotations

1. GAG release levels in the chondrocyte secretome upon cytokine exposure

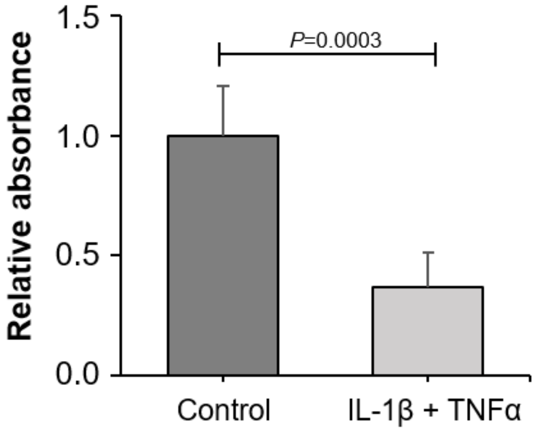

Figure S1. GAG release levels in the chondrocyte secretome upon cytokine exposure (IL-1β+TNF-α, both 10 ng/mL) versus control conditions. GAG release as measured by DMMB assay shows a significant reduction of GAG in the chondrocyte secretome upon cytokine exposure as compared to control. Measurements from four horses (four biological replicates) were combined to provide final values for each group (mean ± SD).

2. Apoptotic rate in primary chondrocytes exposed to pro-inflammatory cytokines

Primary equine articular chondrocytes in the experimental group were treated with the pro-inflammatory cytokines IL-1β and TNF-α (both at 10 ng/mL) (equine recombinant, R&D Systems, Minneapolis, MN, USA) for 7 days. Rate of apoptosis was measured using the luminescence based Promega Caspase-Glo 3/7 Assay System, by determining caspase 3 and 7 activation, according to the instructions of the manufacturer. Luminescence was detected using a Tecan SPARK 10M Plate Reader (Tecan, Männedorf, Switzerland).

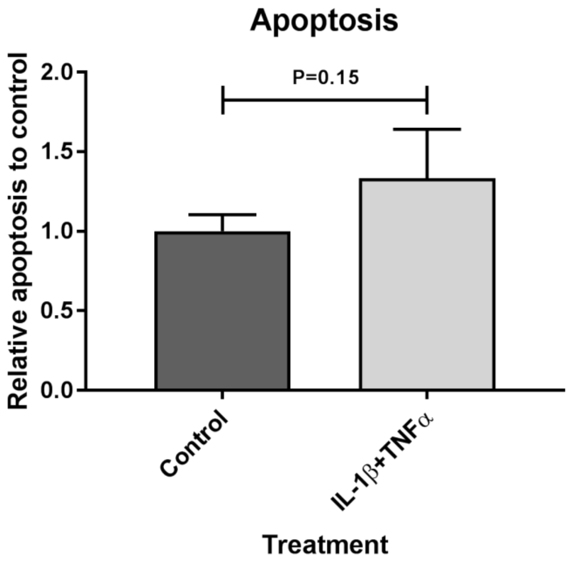

Figure S2. Rate of apoptosis of chondrocytes under inflammatory (IL-1β+TNF-α, both 10 ng/mL) versus control conditions. Apoptotic rate was unchanged under inflammatory conditions (P=0.15).

Table S1. List of proteins classified as *Enzymes* based on GO annotations

Accession	Entry name	Description	Sample
Q05927	5NTD_BOVIN	5'-nucleotidase (5'-NT) (EC 3.1.3.5) (Ecto-5'-nucleotidase) (CD antigen CD73)	Both
P08907	AATM_HORSE	Aspartate aminotransferase, mitochondrial (mAspAT) (EC 2.6.1.1) (EC 2.6.1.7) (Fatty acid-binding protein) (FABP-1)	Control
Q10741	ADA10_BOVIN	Disintegrin and metalloproteinase domain-containing protein 10 (ADAM 10) (EC 3.4.24.81) (CD antigen CD156c)	Both
P04075	ALDOA_HUMAN	Fructose-bisphosphate aldolase A (EC 4.1.2.13) (Lung cancer antigen NY-LU-1) (Muscle-type aldolase)	Cytokine
P09972	ALDOC_HUMAN	Fructose-bisphosphate aldolase C (EC 4.1.2.13) (Brain-type aldolase)	Control
P84085	ARF5_HUMAN	ADP-ribosylation factor 5	Control
P62330	ARF6_HUMAN	ADP-ribosylation factor 6	Control
P05089	ARGI1_HUMAN	Arginase-1 (EC 3.5.3.1) (Liver-type arginase) (Type I arginase)	Control
P58242	ASM3B_MOUSE	Acid sphingomyelinase-like phosphodiesterase 3b (ASM-like phosphodiesterase 3b) (EC 3.1.4.-)	Cytokine
Q28056	ASPH_BOVIN	Aspartyl/asparaginyl beta-hydroxylase (EC 1.14.11.16) (Aspartate beta-hydroxylase) (ASP beta-hydroxylase) (Peptide-aspartate beta-dioxygenase)	Control
P23634	AT2B4_HUMAN	Plasma membrane calcium-transporting ATPase 4 (PMCA4) (EC 7.2.2.10) (Matrix-remodeling-associated protein 1)	Cytokine
Q8TF62	AT8B4_HUMAN	Probable phospholipid-transporting ATPase IM (EC 7.6.2.1) (ATPase class I type 8B member 4) (P4-ATPase flippase complex alpha subunit ATP8B4)	Control
P19483	ATPA_BOVIN	ATP synthase subunit alpha, mitochondrial (ATP synthase F1 subunit alpha)	Control
P11021	BIP_HUMAN	Endoplasmic reticulum chaperone BiP (EC 3.6.4.10) (78 kDa glucose-regulated protein) (GRP-78) (Binding-immunoglobulin protein)	Both
P48754	BRCA1_MOUSE	Breast cancer type 1 susceptibility protein homolog (EC 2.3.2.27) (RING-type E3 ubiquitin transferase BRCA1)	Cytokine
P11586	C1TC_HUMAN	C-1-tetrahydrofolate synthase, cytoplasmic (C1-THF synthase)	Control
P07384	CAN1_HUMAN	Calpain-1 catalytic subunit (EC 3.4.22.52) (Calcium-activated neutral proteinase 1) (CANP 1) (Cell proliferation-inducing gene 30 protein)	Control
P17655	CAN2_HUMAN	Calpain-2 catalytic subunit (EC 3.4.22.53) (Calcium-activated neutral proteinase 2) (CANP 2)	Control
P80209	CATD_BOVIN	Cathepsin D (EC 3.4.23.5)	Control
P14384	CBPM_HUMAN	Carboxypeptidase M (CPM) (EC 3.4.17.12)	Both
Q2KJ93	CDC42_BOVIN	Cell division control protein 42 homolog	Control
Q9UHN6	CEIP2_HUMAN	Cell surface hyaluronidase (EC 3.2.1.35) (Cell migration-inducing hyaluronidase 2) (Transmembrane protein 2)	Cytokine
Q8WUJ3	CEMIP_HUMAN	Cell migration-inducing and hyaluronan-binding protein (EC 3.2.1.35)	Control
P00450	CERU_HUMAN	Ceruloplasmin (EC 1.16.3.1) (Ferroxidase)	Both
P06623	CN37_BOVIN	2',3'-cyclic-nucleotide 3'-phosphodiesterase (CNP) (CNPase) (EC 3.1.4.37)	Control
P06813	CPNS1_RABIT	Calpain small subunit 1 (CSS1) (Calcium-activated neutral proteinase small subunit) (CANP small subunit)	Control
P81605	DCD_HUMAN	Dermcidin (EC 3.4.-.-) (Preproteolysin)	Both
O75912	DGKI_HUMAN	Diacylglycerol kinase iota (DAG kinase iota) (EC 2.7.1.107) (Diglyceride kinase iota) (DGK-iota)	Control
P42891	ECE1_BOVIN	Endothelin-converting enzyme 1 (ECE-1) (EC 3.4.24.71)	Both
A2Q0Z0	EF1A1_HORSE	Elongation factor 1-alpha 1 (EF-1-alpha-1) (Elongation factor Tu) (EF-Tu) (Eukaryotic elongation factor 1 A-1) (eEF1A-1)	Both
Q5VTE0	EF1A3_HUMAN	Putative elongation factor 1-alpha-like 3 (EF-1-alpha-like 3) (Eukaryotic elongation factor 1 A-like 3) (eEF1A-like 3)	Both
P13639	EF2_HUMAN	Elongation factor 2 (EF-2)	Both
Q9NZN4	EHD2_HUMAN	EH domain-containing protein 2 (PAST homolog 2)	Control
O94919	ENDD1_HUMAN	Endonuclease domain-containing 1 protein (EC 3.1.30.-)	Both
Q9XSJ4	ENOA_BOVIN	Alpha-enolase (EC 4.2.1.11) (2-phospho-D-glycerate hydro-lyase) (Enolase 1) (HAP47) (Non-neural enolase) (NNE) (Phosphopyruvate hydratase)	Both

Table S1. (continued)

Accession	Entry name	Description	Sample
Q04609	FOLH1_HUMAN	Glutamate carboxypeptidase 2 (EC 3.4.17.21) (Cell growth-inhibiting gene 27 protein) (Folate hydrolase 1) (Folylpoly-gamma-glutamate carboxypeptidase) (FGCP)	Control
Q6PB93	GALT2_MOUSE	Polypeptide N-acetylgalactosaminyltransferase 2 (EC 2.4.1.41) (Polypeptide GalNAc transferase 2)	Cytokine
Q4R592	GNAI2_MACFA	Guanine nucleotide-binding protein G(i) subunit alpha-2 (Adenylate cyclase-inhibiting G alpha protein)	Both
P38646	GRP75_HUMAN	Stress-70 protein, mitochondrial (75 kDa glucose-regulated protein)	Both
Q8TCT9	HM13_HUMAN	Minor histocompatibility antigen H13 (EC 3.4.23.-) (Signal peptide peptidase)	Both
Q9GKX7	HS90A_HORSE	Heat shock protein HSP 90-alpha	Control
P17066	HSP76_HUMAN	Heat shock 70 kDa protein 6 (Heat shock 70 kDa protein B')	Control
A2Q0Z1	HSP7C_HORSE	Heat shock cognate 71 kDa protein (Heat shock 70 kDa protein 8)	Both
P60842	IF4A1_HUMAN	Eukaryotic initiation factor 4A-I (eIF-4A-I) (eIF4A-I) (EC 3.6.4.13) (ATP-dependent RNA helicase eIF4A-1)	Both
P14618	KPYM_HUMAN	Pyruvate kinase PKM (EC 2.7.1.40) (Cytosolic thyroid hormone-binding protein) (CTHBP)	Both
Q9UIQ6	LCAP_HUMAN	Leucyl-cystinyl aminopeptidase (Cystinyl aminopeptidase) (EC 3.4.11.3) (Insulin-regulated membrane aminopeptidase)	Both
Q8WWY8	LIPH_HUMAN	Lipase member H (LIPH) (EC 3.1.1.-) (LPD lipase-related protein) (Membrane-associated phosphatidic acid-selective phospholipase A1-alpha)	Control
P17897	LYZ1_MOUSE	Lysozyme C-1 (EC 3.2.1.17) (1,4-beta-N-acetylmuramidase C) (Lysozyme C type P)	Cytokine
P08905	LYZ2_MOUSE	Lysozyme C-2 (EC 3.2.1.17) (1,4-beta-N-acetylmuramidase C) (Lysozyme C type M)	Cytokine
Q9GLE4	MMP14_BOVIN	Matrix metalloproteinase-14 (MMP-14) (EC 3.4.24.80) (Membrane-type matrix metalloproteinase 1) (MT-MMP 1)	Both
O15439	MRP4_HUMAN	Multidrug resistance-associated protein 4 (ATP-binding cassette sub-family C member 4) (MRP/cMOAT-related ABC transporter)	Both
P08473	NEP_HUMAN	Neprilysin (EC 3.4.24.11) (Atriopeptidase) (Common acute lymphocytic leukemia antigen) (Neutral endopeptidase 24.11) (CD antigen CD10)	Both
Q28389	PAG_HORSE	Pregnancy-associated glycoprotein (PAG) (EC 3.4.23.-)	Control
P05307	PDIA1_BOVIN	Protein disulfide-isomerase (PDI) (EC 5.3.4.1) (Cellular thyroid hormone-binding protein) (Prolyl 4-hydroxylase subunit beta) (p55)	Both
P38657	PDIA3_BOVIN	Protein disulfide-isomerase A3 (EC 5.3.4.1) (58 kDa glucose-regulated protein)	Both
Q29RV1	PDIA4_BOVIN	Protein disulfide-isomerase A4 (EC 5.3.4.1)	Cytokine
Q15084	PDIA6_HUMAN	Protein disulfide-isomerase A6 (EC 5.3.4.1) (Endoplasmic reticulum protein 5)	Cytokine
P00791	PEPA_PIG	Pepsin A (EC 3.4.23.1)	Cytokine
P12273	PIP_HUMAN	Prolactin-inducible protein (Gross cystic disease fluid protein 15) (GCDFP-15) (Prolactin-induced protein) (Secretory actin-binding protein)	Both
Q02809	PLOD1_HUMAN	Procollagen-lysine,2-oxoglutarate 5-dioxygenase 1 (EC 1.14.11.4) (Lysyl hydroxylase 1) (LH1)	Both
O00469	PLOD2_HUMAN	Procollagen-lysine,2-oxoglutarate 5-dioxygenase 2 (EC 1.14.11.4) (Lysyl hydroxylase 2) (LH2)	Both
O14495	PLPP3_HUMAN	Phospholipid phosphatase 3 (EC 3.1.3.4) (Lipid phosphate phosphohydrolase 3) (PAP2-beta) (Phosphatidate phosphohydrolase type 2b)	Both
Q06830	PRDX1_HUMAN	Peroxiredoxin-1 (EC 1.11.1.15) (Natural killer cell-enhancing factor A) (Proliferation-associated gene protein) (Thioredoxin peroxidase 2)	Cytokine
P55786	PSA_HUMAN	Puromycin-sensitive aminopeptidase (PSA) (EC 3.4.11.14) (Cytosol alanyl aminopeptidase) (AAP-S)	Cytokine
Q9CWJ9	PUR9_MOUSE	Bifunctional purine biosynthesis protein PURH	Cytokine
P61106	RAB14_HUMAN	Ras-related protein Rab-14	Both
P62820	RAB1A_HUMAN	Ras-related protein Rab-1A (YPT1-related protein)	Both
Q9H0U4	RAB1B_HUMAN	Ras-related protein Rab-1B	Both
P51156	RAB26_RAT	Ras-related protein Rab-26	Cytokine
Q9BZG1	RAB34_HUMAN	Ras-related protein Rab-34 (Ras-related protein Rab-39) (Ras-related protein Rah)	Both
Q15286	RAB35_HUMAN	Ras-related protein Rab-35 (GTP-binding protein RAY) (Ras-related protein Rab-1C)	Cytokine
Q06AU3	RAB3A_PIG	Ras-related protein Rab-3A	Cytokine

Table S1. (continued)

Accession	Entry name	Description	Sample
Q5KTJ7	RAB3B_MESAU	Ras-related protein Rab-3B	Cytokine
Q86YS6	RAB43_HUMAN	Ras-related protein Rab-43 (Ras-related protein Rab-41)	Cytokine
Q8CB87	RAB44_MOUSE	Ras-related protein Rab-44	Cytokine
P61017	RAB4B_CANLF	Ras-related protein Rab-4B	Cytokine
P51148	RAB5C_HUMAN	Ras-related protein Rab-5C (L1880) (RAB5L)	Control
P35279	RAB6A_MOUSE	Ras-related protein Rab-6A (Rab-6)	Cytokine
P51149	RAB7A_HUMAN	Ras-related protein Rab-7a	Control
A4FV54	RAB8A_BOVIN	Ras-related protein Rab-8A	Cytokine
Q2HJI8	RAB8B_BOVIN	Ras-related protein Rab-8B	Cytokine
P62998	RAC1_BOVIN	Ras-related C3 botulinum toxin substrate 1 (p21-Rac1)	Control
P62833	RAP1A_BOVIN	Ras-related protein Rap-1A (GTP-binding protein smg p21A)	Control
P61223	RAP1B_BOVIN	Ras-related protein Rap-1b (GTP-binding protein smg p21B)	Control
Q99P58	RB27B_MOUSE	Ras-related protein Rab-27B	Cytokine
Q9HB40	RISC_HUMAN	Retinoid-inducible serine carboxypeptidase (EC 3.4.16.-) (Serine carboxypeptidase 1)	Control
A6NIZ1	RP1BL_HUMAN	Ras-related protein Rap-1b-like protein	Control
Q7KZF4	SND1_HUMAN	Staphylococcal nuclease domain-containing protein 1 (EC 3.1.31.1) (100 kDa coactivator)	Control
Q658P3	STEA3_HUMAN	Metalloreductase STEAP3 (EC 1.16.1.-) (Dudulin-2) (Six-transmembrane epithelial antigen of prostate 3)	Both
P22735	TGM1_HUMAN	Protein-glutamine gamma-glutamyltransferase K (EC 2.3.2.13) (Transglutaminase K)	Cytokine
O97508	THIO_HORSE	Thioredoxin (Trx)	Both
Q96JJ7	TMX3_HUMAN	Protein disulfide-isomerase TMX3 (EC 5.3.4.1) (Thioredoxin domain-containing protein 10)	Control
P02788	TRFL_HUMAN	Lactotransferrin (Lactoferrin) (EC 3.4.21.-) (Growth-inhibiting protein 12) (Talalactoferrin)	Control
P36406	TRI23_HUMAN	E3 ubiquitin-protein ligase TRIM23 (EC 2.3.2.27) (ADP-ribosylation factor domain-containing protein 1)	Control
P00760	TRY1_BOVIN	Cationic trypsin (EC 3.4.21.4) (Beta-trypsin)	Cytokine
Q8NBS9	TXND5_HUMAN	Thioredoxin domain-containing protein 5 (Endoplasmic reticulum resident protein 46)	Control
Q5T4S7	UBR4_HUMAN	E3 ubiquitin-protein ligase UBR4 (EC 2.3.2.27) (600 kDa retinoblastoma protein-associated factor)	Both
O60701	UGDH_HUMAN	UDP-glucose 6-dehydrogenase (UDP-Glc dehydrogenase) (UDP-GlcDH) (UDPGDH) (EC 1.1.1.22)	Control
Q9NYU2	UGGG1_HUMAN	UDP-glucose:glycoprotein glucosyltransferase 1 (UGT1) (hUGT1) (EC 2.4.1.-) (UDP--Glc:glycoprotein glucosyltransferase)	Control
Q16851	UGPA_HUMAN	UTP--glucose-1-phosphate uridylyltransferase (EC 2.7.7.9) (UDP-glucose pyrophosphorylase) (UDPGP) (UGPase)	Control

Table S2. List of proteins classified as *Receptors* based on GO annotations

Accession	Entry name	Description	Sample
P30379	1B01_GORGO	Class I histocompatibility antigen, Gogo-B*0101 alpha chain	Control
P30380	1B02_GORGO	Class I histocompatibility antigen, Gogo-B*0102 alpha chain	Control
P30381	1B03_GORGO	Class I histocompatibility antigen, Gogo-B*0103 alpha chain	Control
Q31612	1B73_HUMAN	HLA class I histocompatibility antigen, B-73 alpha chain (MHC class I antigen B*73)	Control
O43707	ACTN4_HUMAN	Alpha-actinin-4 (Non-muscle alpha-actinin 4)	Cytokine
P78536	ADA17_HUMAN	Disintegrin and metalloproteinase domain-containing protein 17 (ADAM 17) (EC 3.4.24.86) (CD antigen CD156b)	Both
Q61072	ADAM9_MOUSE	Disintegrin and metalloproteinase domain-containing protein 9 (ADAM 9) (EC 3.4.24.-) (Meltrin-gamma)	Both
Q8CJ12	AGRG2_MOUSE	Adhesion G-protein coupled receptor G2 (G-protein coupled receptor 64) (Mouse epididymis-specific protein 6) (Me6)	Control
P30533	AMRP_HUMAN	Alpha-2-macroglobulin receptor-associated protein (Alpha-2-MRAP) (Low density lipoprotein receptor-related protein-associated protein 1) (RAP)	Both
P20594	ANPRB_HUMAN	Atrial natriuretic peptide receptor 2 (EC 4.6.1.2) (Atrial natriuretic peptide receptor type B) (ANP-B) (ANPR-B) (NPR-B) (Guanylate cyclase B) (GC-B)	Control
Q9CZ52	ANTR1_MOUSE	Anthrax toxin receptor 1 (Tumor endothelial marker 8)	Both
P79134	ANXA6_BOVIN	Annexin A6 (Annexin VI) (Annexin-6)	Both
Q03247	APOE_BOVIN	Apolipoprotein E (Apo-E)	Both
Q92888	ARHG1_HUMAN	Rho guanine nucleotide exchange factor 1 (115 kDa guanine nucleotide exchange factor) (p115-RhoGEF) (p115RhoGEF) (Sub1.5)	Control
Q9WU60	ATRN_MOUSE	Attractin (Protein mahogany)	Control
P18075	BMP7_HUMAN	Bone morphogenetic protein 7 (BMP-7) (Osteogenic protein 1) (OP-1) (Eptotermin alfa)	Cytokine
P34821	BMP8A_MOUSE	Bone morphogenetic protein 8A (BMP-8A) (Osteogenic protein 2) (OP-2)	Cytokine
P55105	BMP8B_MOUSE	Bone morphogenetic protein 8B (BMP-8B)	Cytokine
Q9BY67	CADM1_HUMAN	Cell adhesion molecule 1 (Immunoglobulin superfamily member 4) (IgSF4) (Nectin-like protein 2) (NECL-2)	Both
P28491	CALR_PIG	Calreticulin (CRP55) (Calregulin) (Endoplasmic reticulum resident protein 60) (ERp60) (HACBP)	Both
P27824	CALX_HUMAN	Calnexin (IP90) (Major histocompatibility complex class I antigen-binding protein p88) (p90)	Control
Q3ZBH3	CD151_BOVIN	CD151 antigen (CD antigen CD151)	Both
Q13740	CD166_BOVIN	CD166 antigen (Activated leukocyte cell adhesion molecule) (CD antigen CD166)	Both
Q5ZPR3	CD276_HUMAN	CD276 antigen (4Ig-B7-H3) (B7 homolog 3) (B7-H3) (Costimulatory molecule) (CD antigen CD276)	Both
Q05078	CD44_HORSE	CD44 antigen (Extracellular matrix receptor III) (ECMR-III) (GP90 lymphocyte homing/adhesion receptor) (Hyaluronate receptor)	Both
Q9N0K1	CD47_BOVIN	Leukocyte surface antigen CD47 (Integrin-associated protein) (IAP) (CD antigen CD47)	Both
Q29482	CLUS_HORSE	Clusterin [Cleaved into: Clusterin beta chain; Clusterin alpha chain]	Both
P02461	CO3A1_HUMAN	Collagen alpha-1(III) chain	Both
Q8K4Q8	COL12_MOUSE	Collectin-12 (Collectin placenta protein 1) (CL-P1) (Scavenger receptor with C-type lectin)	Cytokine
O75131	CPNE3_HUMAN	Copine-3 (Copine III)	Control
Q16832	DDR2_HUMAN	Discoidin domain-containing receptor 2 (Discoidin domain receptor 2) (EC 2.7.10.1) (CD167 antigen-like family member B)	Both
Q12959	DLG1_HUMAN	Disks large homolog 1 (Synapse-associated protein 97) (SAP-97) (SAP97) (hDlg)	Control
Q15700	DLG2_HUMAN	Disks large homolog 2 (Channel-associated protein of synapse-110) (Chapsyn-110) (Postsynaptic density protein PSD-93)	Control
O43854	EDIL3_HUMAN	EGF-like repeat and discoidin I-like domain-containing protein 3 (Developmentally-regulated endothelial cell locus 1 protein) (Integrin-binding protein DEL1)	Both
P98172	EFNB1_HUMAN	Ephrin-B1 (EFL-3) (ELK ligand) (ELK-L) (EPH-related receptor tyrosine kinase ligand 2) (LERK-2)	Both

Table S2. (continued)

Accession	Entry name	Description	Sample
P00533	EGFR_HUMAN	Epidermal growth factor receptor (EC 2.7.10.1) (Proto-oncogene c-ErbB-1) (Receptor tyrosine-protein kinase erbB-1)	Both
P17813	EGLN_HUMAN	Endoglin (CD antigen CD105)	Both
P14625	ENPL_HUMAN	Endoplasmin (94 kDa glucose-regulated protein) (GRP-94) (Heat shock protein 90 kDa beta member 1) (Tumor rejection antigen 1) (gp96 homolog)	Both
P22413	ENPP1_HUMAN	Ectonucleotide pyrophosphatase/phosphodiesterase family member 1 (E-NPP 1) (Membrane component chromosome 6 surface marker 1)	Both
Q5R5M5	ENPP3_PONAB	Ectonucleotide pyrophosphatase/phosphodiesterase family member 3 (E-NPP 3) (Phosphodiesterase I beta) (PD-Ibeta)	Control
P29317	EPHA2_HUMAN	Ephrin type-A receptor 2 (EC 2.7.10.1) (Epithelial cell kinase) (Tyrosine-protein kinase receptor ECK)	Both
P54764	EPHA4_HUMAN	Ephrin type-A receptor 4 (EC 2.7.10.1) (EPH-like kinase 8) (EK8) (hEK8) (Tyrosine-protein kinase TYRO1) (Tyrosine-protein kinase receptor SEK)	Both
P54756	EPHA5_HUMAN	Ephrin type-A receptor 5 (EC 2.7.10.1) (Brain-specific kinase) (EPH homology kinase 1) (EHK-1) (EPH-like kinase 7) (EK7) (hEK7)	Control
O09127	EPHA8_MOUSE	Ephrin type-A receptor 8 (EC 2.7.10.1) (EPH- and ELK-related kinase) (Tyrosine-protein kinase receptor EEK)	Control
Q5JZY3	EPHAA_HUMAN	Ephrin type-A receptor 10 (EC 2.7.10.1)	Control
P54761	EPHB4_MOUSE	Ephrin type-B receptor 4 (EC 2.7.10.1) (Developmental kinase 2) (mDK-2) (Hepatoma transmembrane kinase) (Tyrosine kinase MYK-1)	Both
Q61851	FGFR3_MOUSE	Fibroblast growth factor receptor 3 (FGFR-3) (EC 2.7.10.1) (Heparin-binding growth factor receptor) (CD antigen CD333)	Control
P21333	FLNA_HUMAN	Filamin-A (FLN-A) (Actin-binding protein 280) (ABP-280) (Alpha-filamin) (Endothelial actin-binding protein) (Filamin-1) (Non-muscle filamin)	Both
O43155	FLRT2_HUMAN	Leucine-rich repeat transmembrane protein FLRT2 (Fibronectin-like domain-containing leucine-rich transmembrane protein 2)	Both
P02702	FOLR1_BOVIN	Folate receptor alpha (FR-alpha) (Folate receptor 1) (Folate-binding protein 1) (FBP) (Milk folate-binding protein)	Cytokine
P14207	FOLR2_HUMAN	Folate receptor beta (FR-beta) (Folate receptor 2) (Folate receptor, fetal/placental) (Placental folate-binding protein) (FBP)	Cytokine
Q9UP38	FZD1_HUMAN	Frizzled-1 (Fz-1) (hFz1) (FzE1)	Both
Q14332	FZD2_HUMAN	Frizzled-2 (Fz-2) (hFz2) (FzE2)	Both
O75084	FZD7_HUMAN	Frizzled-7 (Fz-7) (hFz7) (FzE3)	Both
P04899	GNAI2_HUMAN	Guanine nucleotide-binding protein G(i) subunit alpha-2 (Adenylate cyclase-inhibiting G alpha protein)	Both
P08754	GNAI3_HUMAN	Guanine nucleotide-binding protein G(k) subunit alpha (G(i) alpha-3)	Control
Q9Y625	GPC6_HUMAN	Glypican-6 [Cleaved into: Secreted glypican-6]	Control
P32942	ICAM3_HUMAN	Intercellular adhesion molecule 3 (ICAM-3) (CDw50) (ICAM-R) (CD antigen CD50)	Control
P08069	IGF1R_HUMAN	Insulin-like growth factor 1 receptor (EC 2.7.10.1) (Insulin-like growth factor I receptor) (IGF-I receptor) (CD antigen CD221)	Both
Q14005	IL16_HUMAN	Pro-interleukin-16 [Cleaved into: Interleukin-16 (IL-16) (Lymphocyte chemoattractant factor) (LCF)]	Control
P40189	IL6RB_HUMAN	Interleukin-6 receptor subunit beta (IL-6 receptor subunit beta) (IL-6R subunit beta) (IL-6R-beta) (CD antigen CD130)	Control
Q14974	IMB1_HUMAN	Importin subunit beta-1 (Importin-90) (Karyopherin subunit beta-1) (Nuclear factor p97) (Pore targeting complex 97 kDa subunit) (PTAC97)	Both
P06213	INSR_HUMAN	Insulin receptor (IR) (EC 2.7.10.1) (CD antigen CD220) [Cleaved into: Insulin receptor subunit alpha; Insulin receptor subunit beta]	Cytokine
P56199	ITA1_HUMAN	Integrin alpha-1 (CD49 antigen-like family member A) (Laminin and collagen receptor) (VLA-1) (CD antigen CD49a)	Both
O75578	ITA10_HUMAN	Integrin alpha-10	Control
Q9UKX5	ITA11_HUMAN	Integrin alpha-11	Both
F1MMS9	ITA3_BOVIN	Integrin alpha-3 (CD49 antigen-like family member C) (Galactoprotein B3) (GAPB3) (VLA-3 subunit alpha) (CD antigen CD49c)	Both
Q27977	ITA5_BOVIN	Integrin alpha-5 (Fibronectin receptor subunit alpha) (Integrin alpha-F) (VLA-5)	Both
P23229	ITA6_HUMAN	Integrin alpha-6 (CD49 antigen-like family member F) (VLA-6) (CD antigen CD49f)	Both
P43406	ITAV_MOUSE	Integrin alpha-V (Vitronectin receptor subunit alpha) (CD antigen CD51) [Cleaved into: Integrin alpha-V heavy chain; Integrin alpha-V light chain]	Control
P53712	ITB1_BOVIN	Integrin beta-1 (Fibronectin receptor subunit beta) (VLA-4 subunit beta) (CD antigen CD29)	Both
P32592	ITB2_BOVIN	Integrin beta-2 (Cell surface adhesion glycoproteins LFA-1/CR3/p150,95 subunit beta) (Complement receptor C3 subunit beta) (CD antigen CD18)	Cytokine

Table S2. (continued)

Accession	Entry name	Description	Sample
P80747	ITB5_BOVIN	Integrin beta-5	Both
P26010	ITB7_HUMAN	Integrin beta-7 (Gut homing receptor beta subunit)	Both
P31025	LCN1_HUMAN	Lipocalin-1 (Tear lipocalin) (Tlc) (Tear prealbumin) (TP) (von Ebner gland protein) (VEG protein)	Both
P01131	LDLR_BOVIN	Low-density lipoprotein receptor (LDL receptor)	Both
P61793	LPAR1_MOUSE	Lysophosphatidic acid receptor 1 (LPA receptor 1) (LPA-1) (Lysophosphatidic acid receptor Edg-2) (Rec1.3) (VZG-1)	Both
Q07954	LRP1_HUMAN	Prolow-density lipoprotein receptor-related protein 1 (LRP-1) (Alpha-2-macroglobulin receptor) (A2MR) (Apolipoprotein E receptor) (APOER) (CD antigen CD91)	Both
Q2QLA9	MET_HORSE	Hepatocyte growth factor receptor (HGF receptor) (EC 2.7.10.1) (HGF/SF receptor) (Proto-oncogene c-Met)	Both
Q2HJ49	MOES_BOVIN	Moesin (Membrane-organizing extension spike protein)	Control
P08169	MPRI_BOVIN	Cation-independent mannose-6-phosphate receptor (300 kDa mannose 6-phosphate receptor) (Insulin-like growth factor 2 receptor) (CD antigen CD222)	Control
P35579	MYH9_HUMAN	Myosin-9 (Cellular myosin heavy chain, type A) (Myosin heavy chain 9) (Myosin heavy chain, non-muscle IIa)	Control
Q15223	NECT1_HUMAN	Nectin-1 (Herpes virus entry mediator C) (Nectin cell adhesion molecule 1) (CD antigen CD111)	Both
Q9NQS3	NECT3_HUMAN	Nectin-3 (CDw113) (Nectin cell adhesion molecule 3) (Poliovirus receptor-related protein 3) (CD antigen CD113)	Both
Q8NFZ4	NLGN2_HUMAN	Neuroligin-2	Control
Q04721	NOTC2_HUMAN	Neurogenic locus notch homolog protein 2 (Notch 2)	Both
Q9Y639	NPTN_HUMAN	Neuroplastin (Stromal cell-derived receptor 1) (SDR-1)	Both
O14786	NRP1_HUMAN	Neuropilin-1 (Vascular endothelial cell growth factor 165 receptor) (CD antigen CD304)	Cytokine
O60462	NRP2_HUMAN	Neuropilin-2 (Vascular endothelial cell growth factor 165 receptor 2)	Both
P16234	PGFRA_HUMAN	Platelet-derived growth factor receptor alpha (PDGF-R-alpha) (PDGFR-alpha) (EC 2.7.10.1) (Alpha platelet-derived growth factor receptor) (CD antigen CD140a)	Control
P09619	PGFRB_HUMAN	Platelet-derived growth factor receptor beta (PDGF-R-beta) (PDGFR-beta) (EC 2.7.10.1) (CD antigen CD140b)	Both
Q8SPJ1	PLAK_BOVIN	Junction plakoglobin (Desmoplakin III) (Desmoplakin-3)	Both
Q9UIW2	PLXA1_HUMAN	Plexin-A1 (Semaphorin receptor NOV)	Both
O43157	PLXB1_HUMAN	Plexin-B1 (Semaphorin receptor SEP)	Cytokine
O15031	PLXB2_HUMAN	Plexin-B2 (MM1)	Both
P49927	PRIO_PIG	Major prion protein (PrP) (CD antigen CD230)	Cytokine
Q1LZF7	PTH1R_BOVIN	Parathyroid hormone/parathyroid hormone-related peptide receptor (PTH/PTHrP type I receptor) (PTH1 receptor)	Control
Q13308	PTK7_HUMAN	Inactive tyrosine-protein kinase 7 (Colon carcinoma kinase 4)	Both
P18433	PTPRA_HUMAN	Receptor-type tyrosine-protein phosphatase alpha (Protein-tyrosine phosphatase alpha) (R-PTP-alpha) (EC 3.1.3.48)	Both
P23470	PTPRG_HUMAN	Receptor-type tyrosine-protein phosphatase gamma (Protein-tyrosine phosphatase gamma) (R-PTP-gamma) (EC 3.1.3.48)	Both
Q12913	PTPRJ_HUMAN	Receptor-type tyrosine-protein phosphatase eta (Protein-tyrosine phosphatase eta) (R-PTP-eta) (EC 3.1.3.48) (CD antigen CD148)	Control
P28828	PTPRM_MOUSE	Receptor-type tyrosine-protein phosphatase mu (Protein-tyrosine phosphatase mu) (R-PTP-mu) (EC 3.1.3.48)	Control
Q92626	PXDN_HUMAN	Peroxidasin homolog (EC 1.11.1.7) (Melanoma-associated antigen MG50) (Vascular peroxidase 1) (p53-responsive gene 2 protein)	Cytokine
P20338	RAB4A_HUMAN	Ras-related protein Rab-4A	Cytokine
O95980	RECK_HUMAN	Reversion-inducing cysteine-rich protein with Kazal motifs (hRECK) (Suppressor of tumorigenicity 15 protein)	Both
Q9Y6N7	ROBO1_HUMAN	Roundabout homolog 1 (Deleted in U twenty twenty) (H-Robo-1)	Both
Q01974	ROR2_HUMAN	Tyrosine-protein kinase transmembrane receptor ROR2 (EC 2.7.10.1) (Neurotrophic tyrosine kinase, receptor-related 2)	Control
P08922	ROS1_HUMAN	Proto-oncogene tyrosine-protein kinase ROS (EC 2.7.10.1) (Proto-oncogene c-Ros)	Control
P31151	S10A7_HUMAN	Protein S100-A7 (Psoriasin) (S100 calcium-binding protein A7)	Both

Table S2. (continued)

Accession	Entry name	Description	Sample
P06702	S10A9_HUMAN	Protein S100-A9 (Calgranulin-B) (Calprotectin L1H subunit) (Leukocyte L1 complex heavy chain)	Cytokine
Q8WUM9	S20A1_HUMAN	Sodium-dependent phosphate transporter 1 (Phosphate transporter 1) (PiT-1) (Solute carrier family 20 member 1)	Both
Q6AZY7	SCAR3_HUMAN	Scavenger receptor class A member 3 (Cellular stress response gene protein)	Control
A5D7B7	SEPR_BOVIN	Prolyl endopeptidase FAP (EC 3.4.21.26) (Dipeptidyl peptidase FAP) (EC 3.4.14.5) (Fibroblast activation protein alpha)	Both
Q2KJH6	SERPH_BOVIN	Serpin H1 (Collagen-binding protein) (Colligin)	Both
Q6PHU5	SORT_MOUSE	Sortilin (Neurotensin receptor 3) (NTR3) (mNTR3)	Control
Q2V905	TFR1_HORSE	Transferrin receptor protein 1 (TR) (TfR) (TfR1) (Trfr) (CD antigen CD71)	Both
Q03167	TGBR3_HUMAN	Transforming growth factor beta receptor type 3 (TGF-beta receptor type 3) (TGFR-3) (Betaglycan)	Control
P37173	TGFR2_HUMAN	TGF-beta receptor type-2 (TGFR-2) (EC 2.7.11.30) (TGF-beta type II receptor)	Control
P04216	THY1_HUMAN	Thy-1 membrane glycoprotein (CDw90) (Thy-1 antigen) (CD antigen CD90)	Control
Q9Y490	TLN1_HUMAN	Talin-1	Both
P24627	TRFL_BOVIN	Lactotransferrin (Lactoferrin) (EC 3.4.21.-) [Cleaved into: Lactoferricin-B (Lfcin-B)]	Cytokine
Q28178	TSP1_BOVIN	Thrombospondin-1 (Glycoprotein G)	Both
P30530	UFO_HUMAN	Tyrosine-protein kinase receptor UFO (EC 2.7.10.1) (AXL oncogene)	Both
Q28260	VCAM1_CANLF	Vascular cell adhesion protein 1 (V-CAM 1) (VCAM-1) (CD antigen CD106)	Both
P98156	VLDLR_MOUSE	Very low-density lipoprotein receptor (VLDL receptor) (VLDL-R)	Control
Q2HJG5	VPS35_BOVIN	Vacuolar protein sorting-associated protein 35 (Vesicle protein sorting 35)	Both
Q9EQH3	VPS35_MOUSE	Vacuolar protein sorting-associated protein 35 (Maternal-embryonic 3) (Vesicle protein sorting 35)	Control
P04004	VTNC_HUMAN	Vitronectin (VN) (S-protein) (Serum-spreading factor) (V75) [Cleaved into: Vitronectin V65 subunit; Vitronectin V10 subunit; Somatomedin-B]	Control

Table S3. List of proteins classified as *Transporters* based on GO annotations

Accession	Entry name	Description	Sample
P62258	1433E_HUMAN	14-3-3 protein epsilon (14-3-3E)	Both
P08195	4F2_HUMAN	4F2 cell-surface antigen heavy chain (4F2hc) (4F2 heavy chain antigen) (Solute carrier family 3 member 2) (CD antigen CD98)	Both
Q95JC7	AAAT_BOVIN	Neutral amino acid transporter B(0) (ATB(0)) (Sodium-dependent neutral amino acid transporter type 2) (Solute carrier family 1 member 5)	Both
Q4KMQ2	ANO6_HUMAN	Anoctamin-6 (Small-conductance calcium-activated nonselective cation channel) (SCAN channel) (Transmembrane protein 16F)	Both
P04272	ANXA2_BOVIN	Annexin A2 (Annexin II) (Annexin-2)(Calpactin-1 heavy chain) (Chromobindin-8) (Lipocortin II)	Both
P14824	ANXA6_MOUSE	Annexin A6 (67 kDa calelectrin) (Annexin VI) (Annexin-6) (Calphobindin-II) (Chromobindin-20) (Lipocortin VI)	Cytokine
P54707	AT12A_HUMAN	Potassium-transporting ATPase alpha chain 2 (EC 7.2.2.19) (Non-gastric H(+)/K(+) ATPase subunit alpha) (Proton pump)	Cytokine
P18907	AT1A1_HORSE	Sodium/potassium-transporting ATPase subunit alpha-1 (Sodium pump subunit alpha-1) (EC 7.2.2.13) (Na(+)/K(+) ATPase alpha-1 subunit)	Both
Q13733	AT1A4_HUMAN	Sodium/potassium-transporting ATPase subunit alpha-4 (Na(+)/K(+) ATPase alpha-4 subunit) (EC 7.2.2.13) (Sodium pump subunit alpha-4)	Both
P20020	AT2B1_HUMAN	Plasma membrane calcium-transporting ATPase 1 (EC 7.2.2.10) (Plasma membrane calcium ATPase isoform 1) (PMCA1) (Plasma membrane calcium pump isoform 1)	Both
P06576	ATPB_HUMAN	ATP synthase subunit beta, mitochondrial (EC 7.1.2.2) (ATP synthase F1 subunit beta)	Control
Q6SJP2	B3A2_HORSE	Anion exchange protein 2 (AE 2) (Anion exchanger 2) (Non-erythroid band 3-like protein) (Solute carrier family 4 member 2)	Both
P00974	BPT1_BOVIN	Pancreatic trypsin inhibitor (Aprotinin) (Basic protease inhibitor) (BPI) (BPTI)	Both
P54289	CA2D1_HUMAN	Voltage-dependent calcium channel subunit alpha-2/delta-1 (Voltage-gated calcium channel subunit alpha-2/delta-1)	Both
O60840	CAC1F_HUMAN	Voltage-dependent L-type calcium channel subunit alpha-1F (Voltage-gated calcium channel subunit alpha Cav1.4)	Control
P0DP24	CALM2_HUMAN	Calmodulin-2	Control
P02666	CASB_BOVIN	Beta-casein [Cleaved into: Casoparan; Antioxidant peptide; Casohypotensin]	Both
Q9NV96	CC50A_HUMAN	Cell cycle control protein 50A (P4-ATPase flippase complex beta subunit TMEM30A) (Transmembrane protein 30A)	Both
Q9XSA7	CLIC4_BOVIN	Chloride intracellular channel protein 4 (Intracellular chloride ion channel protein p64H1)	Control
Q9UBL6	CPNE7_HUMAN	Copine-7 (Copine VII)	Control
P52569	CTR2_HUMAN	Cationic amino acid transporter 2 (CAT-2) (CAT2) (Low affinity cationic amino acid transporter 2) (Solute carrier family 7 member 2)	Cytokine
Q5T3F8	CSCL2_HUMAN	CSC1-like protein 2 (Transmembrane protein 63B)	Control
P56564	EAA1_MOUSE	Excitatory amino acid transporter 1 (Sodium-dependent glutamate/aspartate transporter 1) (Solute carrier family 1 member 3)	Both
P11166	GTR1_HUMAN	Solute carrier family 2, facilitated glucose transporter member 1 (Glucose transporter type 1, erythrocyte/brain) (GLUT-1) (HepG2 glucose transporter)	Both
Q8TDB8	GTR14_HUMAN	Solute carrier family 2, facilitated glucose transporter member 14 (Glucose transporter type 14) (GLUT-14)	Cytokine
Q9XSC2	GTR3_RABIT	Solute carrier family 2, facilitated glucose transporter member 3 (Glucose transporter type 3, brain) (GLUT-3) (Fragment)	Both
P58353	GTR5_BOVIN	Solute carrier family 2, facilitated glucose transporter member 5 (Fructose transporter) (Glucose transporter type 5, small intestine) (GLUT-5)	Control
Q8WN96	ITPR2_BOVIN	Inositol 1,4,5-trisphosphate receptor type 2 (IP3 receptor isoform 2) (IP3R 2) (InsP3R2) (Type 2 inositol 1,4,5-trisphosphate receptor) (Type 2 InsP3 receptor)	Control
Q8IWT6	LRC8A_HUMAN	Volume-regulated anion channel subunit LRRC8A (Leucine-rich repeat-containing protein 8A) (Swelling protein 1)	Cytokine
Q7L1W4	LRC8D_HUMAN	Volume-regulated anion channel subunit LRRC8D (Leucine-rich repeat-containing protein 5) (Leucine-rich repeat-containing protein 8D)	Cytokine
O15427	MOT4_HUMAN	Monocarboxylate transporter 4 (MCT 4) (Solute carrier family 16 member 3)	Both
Q00325	MPCP_HUMAN	Phosphate carrier protein, mitochondrial (Phosphate transport protein) (PTP) (Solute carrier family 25 member 3)	Both
Q8HXQ5	MRP1_BOVIN	Multidrug resistance-associated protein 1 (ATP-binding cassette sub-family C member 1) (Leukotriene C(4) transporter) (LTC4 transporter)	Both
Q63120	MRP2_RAT	Canalicular multispecific organic anion transporter 1 (Canalicular multidrug resistance protein) (Multidrug resistance-associated protein 2)	Cytokine
Q92508	PIEZ1_HUMAN	Piezo-type mechanosensitive ion channel component 1 (Membrane protein induced by beta-amyloid treatment) (Mib) (Protein FAM38A)	Both

Table S3. (continued)

Accession	Entry name	Description	Sample
P62935	PPIA_BOVIN	Peptidyl-prolyl cis-trans isomerase A (PPIase A) (EC 5.2.1.8) (Cyclophilin A)	Both
P60902	S10AA_BOVIN	Protein S100-A10 (Calpactin I light chain) (Calpactin-1 light chain) (Cellular ligand of annexin II) (S100 calcium-binding protein A10)	Control
P55011	S12A2_HUMAN	Solute carrier family 12 member 2 (Basolateral Na-K-Cl symporter) (Bumetanide-sensitive sodium-(potassium)-chloride cotransporter 1)	Both
Q9UP95	S12A4_HUMAN	Solute carrier family 12 member 4 (Electroneutral potassium-chloride cotransporter 1) (Erythroid K-Cl cotransporter 1) (hKCC1)	Both
Q65AC2	S26A2_HORSE	Sulfate transporter (Solute carrier family 26 member 2)	Control
A2VE31	S38A2_BOVIN	Sodium-coupled neutral amino acid transporter 2 (Amino acid transporter A2) (Solute carrier family 38 member 2)	Cytokine
Q5E9S9	S38A5_BOVIN	Sodium-coupled neutral amino acid transporter 5 (Solute carrier family 38 member 5) (System N transporter 2)	Cytokine
A5D7L5	S39AE_BOVIN	Zinc transporter ZIP14 (Solute carrier family 39 member 14) (Zrt- and Irt-like protein 14) (ZIP-14)	Both
Q9Y6R1	S4A4_HUMAN	Electrogenic sodium bicarbonate cotransporter 1 (Sodium bicarbonate cotransporter) (Na(+)/HCO3(-) cotransporter) (Solute carrier family 4 member 4) (kNBC1)	Both
Q8BTY2	S4A7_MOUSE	Sodium bicarbonate cotransporter 3 (Solute carrier family 4 member 7)	Both
P53793	SC5A3_BOVIN	Sodium/myo-inositol cotransporter (Na(+)/myo-inositol cotransporter) (Sodium/myo-inositol transporter 1) (SMIT1) (Solute carrier family 5 member 3)	Both
Q9Y289	SC5A6_HUMAN	Sodium-dependent multivitamin transporter (Na(+)-dependent multivitamin transporter) (Solute carrier family 5 member 6)	Control
P23791	SL9A1_RABIT	Sodium/hydrogen exchanger 1 (Na(+)/H(+) exchanger 1) (NHE-1) (Solute carrier family 9 member 1)	Both
Q92581	SL9A6_HUMAN	Sodium/hydrogen exchanger 6 (Na(+)/H(+) exchanger 6) (NHE-6) (Solute carrier family 9 member 6)	Control
Q9UHE8	STEA1_HUMAN	Metalloreductase STEAP1 (EC 1.16.1.-) (Six-transmembrane epithelial antigen of prostate 1)	Control
Q9Y6M5	ZNT1_HUMAN	Zinc transporter 1 (ZnT-1) (Solute carrier family 30 member 1)	Cytokine

Table S4. List of proteins that could not be classified into any of the previous categories based on GO annotations (listed as *Unclassified)*

Accession	Entry name	Description	Sample
P30461	1B13_HUMAN	HLA class I histocompatibility antigen, B-13 alpha chain (MHC class I antigen B*13)	Control
P34955	A1AT_BOVIN	Alpha-1-antiproteinase (Alpha-1-antitrypsin) (Alpha-1-proteinase inhibitor) (Serpin A1)	Cytokine
Q7SIH1	A2MG_BOVIN	Alpha-2-macroglobulin (Alpha-2-M)	Control
P84336	ACTB_CAMDR	Actin, cytoplasmic 1 (Beta-actin) [Cleaved into: Actin, cytoplasmic 1, N-terminally processed]	Cytokine
Q562R1	ACTBL_HUMAN	Beta-actin-like protein 2 (Kappa-actin)	Control
Q09666	AHNK_HUMAN	Neuroblast differentiation-associated protein AHNAK (Desmoyokin)	Both
P02769	ALBU_BOVIN	Serum albumin (BSA) (allergen Bos d 6)	Both
Q4R4H7	ANXA5_MACFA	Annexin A5 (Annexin V) (Annexin-5)	Both
P13928	ANXA8_HUMAN	Annexin A8 (Annexin VIII) (Annexin-8) (Vascular anticoagulant-beta) (VAC-beta)	Control
Q5E9I6	ARF3_BOVIN	ADP-ribosylation factor 3	Control
Q3SZF2	ARF4_BOVIN	ADP-ribosylation factor 4	Both
Q9UPA5	BSN_HUMAN	Protein bassoon (Zinc finger protein 231)	Control
Q05682	CALD1_HUMAN	Caldesmon (CDM)	Both
O43852	CALU_HUMAN	Calumenin (Crocalbin) (IEF SSP 9302)	Both
P02662	CASA1_BOVIN	Alpha-S1-casein (allergen Bos d 8) [Cleaved into: Antioxidant peptide]	Both
P02663	CASA2_BOVIN	Alpha-S2-casein [Cleaved into: Casocidin-1 (Casocidin-I)]	Both
P02668	CASK_BOVIN	Kappa-casein [Cleaved into: Casoxin-C; Casoxin-6; Casoxin-A; Casoxin-B; Casoplatelin]	Both
Q6YHK3	CD109_HUMAN	CD109 antigen (150 kDa TGF-beta-1-binding protein) (C3 and PZP-like alpha-2-macroglobulin domain-containing protein 7) (CD antigen CD109)	Both
Q27954	COPA_BOVIN	Coatomer subunit alpha (Alpha-coat protein) (Alpha-COP) (HEP-COP) (HEPCOP) [Cleaved into: Xenin (Xenopsin-related peptide); Proxenin]	Control
A0JN39	COPB_BOVIN	Coatomer subunit beta (Beta-coat protein) (Beta-COP)	Cytokine
Q96FN4	CPNE2_HUMAN	Copine-2 (Copine II)	Control
Q96A23	CPNE4_HUMAN	Copine-4 (Copine IV) (Copine-8)	Control
Q9HCH3	CPNE5_HUMAN	Copine-5 (Copine V)	Control
Q2KHY1	CPNE6_BOVIN	Copine-6 (Copine VI)	Control
Q86YQ8	CPNE8_HUMAN	Copine-8 (Copine VIII)	Control
Q8IYJ1	CPNE9_HUMAN	Copine-9 (Copine IX)	Control
Q96CG8	CTHR1_HUMAN	Collagen triple helix repeat-containing protein 1 (Protein NMTC1)	Control
Q6UVK1	CSPG4_HUMAN	Chondroitin sulfate proteoglycan 4 (Chondroitin sulfate proteoglycan NG2)	Both
Q29243	DAG1_PIG	Dystroglycan (Dystrophin-associated glycoprotein 1) [Cleaved into: Alpha-dystroglycan (Alpha-DG); Beta-dystroglycan (Beta-DG)]	Both
Q14204	DYHC1_HUMAN	Cytoplasmic dynein 1 heavy chain 1 (Cytoplasmic dynein heavy chain 1) (Dynein heavy chain, cytosolic)	Both
Q58DR6	EMP3_BOVIN	Epithelial membrane protein 3 (EMP-3)	Both
Q01844	EWS_HUMAN	RNA-binding protein EWS (EWS oncogene) (Ewing sarcoma breakpoint region 1 protein)	Both
Q5M7W6	F234A_RAT	Protein FAM234A (Protein ITFG3)	Control
P12763	FETUA_BOVIN	Alpha-2-HS-glycoprotein (Asialofetuin) (Fetuin-A)	Both
Q9P2B2	FPRP_HUMAN	Prostaglandin F2 receptor negative regulator (CD9 partner 1) (CD9P-1) (Glu-Trp-Ile EWI motif-containing protein F) (CD antigen CD315)	Both

Table S4. (continued)

Accession	Entry name	Description	Sample
P35052	GPC1_HUMAN	Glypican-1 [Cleaved into: Secreted glypican-1]	Control
A2AJ76	HMCN2_MOUSE	Hemicentin-2	Control
Q86YZ3	HORN_HUMAN	Hornerin	Both
Q9GKX8	HS90B_HORSE	Heat shock protein HSP 90-beta	Both
P34932	HSP74_HUMAN	Heat shock 70 kDa protein 4 (HSP70RY) (Heat shock 70-related protein APG-2)	Control
Q9Y4L1	HYOU1_HUMAN	Hypoxia up-regulated protein 1 (150 kDa oxygen-regulated protein) (ORP-150) (170 kDa glucose-regulated protein) (GRP-170)	Both
P13646	K1C13_HUMAN	Keratin, type I cytoskeletal 13 (Cytokeratin-13) (CK-13) (Keratin-13) (K13)	Both
Q04695	K1C17_HUMAN	Keratin, type I cytoskeletal 17 (39.1) (Cytokeratin-17) (CK-17) (Keratin-17) (K17)	Both
P08728	K1C19_BOVIN	Keratin, type I cytoskeletal 19 (Cytokeratin-19) (CK-19) (Keratin-19) (K19)	Both
Q2M2I5	K1C24_HUMAN	Keratin, type I cytoskeletal 24 (Cytokeratin-24) (CK-24) (Keratin-24) (K24) (Type I keratin-24)	Control
P04264	K2C1_HUMAN	Keratin, type II cytoskeletal 1 (67 kDa cytokeratin) (Cytokeratin-1) (CK-1) (Hair alpha protein) (Keratin-1) (K1) (Type-II keratin Kb1)	Both
A5A6M6	K2C1_PANTR	Keratin, type II cytoskeletal 1 (Cytokeratin-1) (CK-1) (Keratin-1) (K1) (Type-II keratin Kb1)	Control
Q7Z794	K2C1B_HUMAN	Keratin, type II cytoskeletal 1b (Cytokeratin-1B) (CK-1B) (Keratin-77) (K77) (Type-II keratin Kb39)	Both
P12035	K2C3_HUMAN	Keratin, type II cytoskeletal 3 (65 kDa cytokeratin) (Cytokeratin-3) (CK-3) (Keratin-3) (K3) (Type-II keratin Kb3)	Cytokine
P19013	K2C4_HUMAN	Keratin, type II cytoskeletal 4 (Cytokeratin-4) (CK-4) (Keratin-4) (K4) (Type-II keratin Kb4)	Both
P48668	K2C6C_HUMAN	Keratin, type II cytoskeletal 6C (Cytokeratin-6C) (CK-6C) (Cytokeratin-6E) (CK-6E) (Keratin K6h) (Keratin-6C) (K6C) (Type-II keratin Kb12)	Both
Q8N1N4	K2C78_HUMAN	Keratin, type II cytoskeletal 78 (Cytokeratin-78) (CK-78) (Keratin-5b) (Keratin-78) (K78) (Type-II keratin Kb40)	Both
P05787	K2C8_HUMAN	Keratin, type II cytoskeletal 8 (Cytokeratin-8) (CK-8) (Keratin-8) (K8) (Type-II keratin Kb8)	Cytokine
Q5T749	KPRP_HUMAN	Keratinocyte proline-rich protein (hKPRP)	Both
P02754	LACB_BOVIN	Beta-lactoglobulin (Beta-LG) (allergen Bos d 5)	Both
P13473	LAMP2_HUMAN	Lysosome-associated membrane glycoprotein 2 (LAMP-2) (CD107 antigen-like family member B) (LGP-96) (CD antigen CD107b)	Both
Q5VSP4	LC1L1_HUMAN	Putative lipocalin 1-like protein 1 (Lipocalin 1-like pseudogene 1)	Both
P49257	LMAN1_HUMAN	Protein ERGIC-53 (ER-Golgi intermediate compartment 53 kDa protein) (Gp58) (Intracellular mannose-specific lectin MR60) (Lectin mannose-binding 1)	Cytokine
P61626	LYSC_HUMAN	Lysozyme C (EC 3.2.1.17) (1,4-beta-N-acetylmuramidase C)	Cytokine
P12067	LYSC1_PIG	Lysozyme C-1 (EC 3.2.1.17) (1,4-beta-N-acetylmuramidase C)	Cytokine
P12069	LYSC3_PIG	Lysozyme C-3 (EC 3.2.1.17) (1,4-beta-N-acetylmuramidase C)	Cytokine
P26042	MOES_PIG	Moesin (Membrane-organizing extension spike protein)	Control
Q32PI9	MPZL1_BOVIN	Myelin protein zero-like protein 1	Control
Q9UKN7	MYO15_HUMAN	Unconventional myosin-XV (Unconventional myosin-15)	Control
Q3SYX0	NDRG1_BOVIN	Protein NDRG1 (N-myc downstream-regulated gene 1 protein)	Control
Q96TA1	NIBL1_HUMAN	Niban-like protein 1 (Meg-3) (Melanoma invasion by ERK) (MINERVA) (Protein FAM129B)	Control
Q02818	NUCB1_HUMAN	Nucleobindin-1 (CALNUC)	Control
Q02819	NUCB1_MOUSE	Nucleobindin-1 (CALNUC)	Both
P80303	NUCB2_HUMAN	Nucleobindin-2 (DNA-binding protein NEFA) (Epididymis secretory protein Li 109) (Gastric cancer antigen Zg4) (Prepronesfatin)	Both
P51178	PLCD1_HUMAN	1-phosphatidylinositol 4,5-bisphosphate phosphodiesterase delta-1 (EC 3.1.4.11) (Phosphoinositide phospholipase C-delta-1)	Both
Q5UJG1	PRIO_ANTCE	Major prion protein (PrP) (CD antigen CD230)	Both
Q16378	PROL4_HUMAN	Proline-rich protein 4 (Lacrimal proline-rich protein) (Nasopharyngeal carcinoma-associated proline-rich protein 4)	Control

Table S4. (continued)

Accession	Entry name	Description	Sample
O77691	S10A6_HORSE	Protein S100-A6 (Calcyclin) (S100 calcium-binding protein A6)	Both
O14828	SCAM3_HUMAN	Secretory carrier-associated membrane protein 3 (Secretory carrier membrane protein 3)	Control
Q58DD4	SDC2_BOVIN	Syndecan-2 (SYND2) (CD antigen CD362)	Cytokine
O75056	SDC3_HUMAN	Syndecan-3 (SYND3)	Both
Q6UXD5	SE6L2_HUMAN	Seizure 6-like protein 2	Control
Q9NRX5	SERC1_HUMAN	Serine incorporator 1 (Tumor differentially expressed protein 1-like) (Tumor differentially expressed protein 2)	Both
Q16585	SGCB_HUMAN	Beta-sarcoglycan (Beta-SG) (43 kDa dystrophin-associated glycoprotein) (43DAG) (A3b)	Control
Q92629	SGCD_HUMAN	Delta-sarcoglycan (Delta-SG) (35 kDa dystrophin-associated glycoprotein) (35DAG)	Both
P81125	SNAA_BOVIN	Alpha-soluble NSF attachment protein (SNAP-alpha) (N-ethylmaleimide-sensitive factor attachment protein alpha)	Control
Q96P63	SPB12_HUMAN	Serpin B12	Control
Q6NZ63	STEAL_HUMAN	STEAP family member 1B	Control
P27105	STOM_HUMAN	Erythrocyte band 7 integral membrane protein (Protein 7.2b) (Stomatin)	Both
Q16563	SYPL1_HUMAN	Synaptophysin-like protein 1 (Pantophysin)	Both
Q5E971	TMEDA_BOVIN	Transmembrane emp24 domain-containing protein 10 (21 kDa transmembrane-trafficking protein)	Control
Q92973	TNPO1_HUMAN	Transportin-1 (Importin beta-2) (Karyopherin beta-2) (M9 region interaction protein) (MIP)	Cytokine
Q7L0X0	TRIL_HUMAN	TLR4 interactor with leucine rich repeats (Leucine-rich repeat-containing protein KIAA0644)	Control
P07477	TRY1_HUMAN	Trypsin-1 (EC 3.4.21.4) (Beta-trypsin) (Cationic trypsinogen) (Serine protease 1) (Trypsin I) [Cleaved into: Alpha-trypsin chain 1; Alpha-trypsin chain 2]	Control
Q32KU6	TSN6_BOVIN	Tetraspanin-6 (Tspan-6)	Control
P0CG48	UBC_HUMAN	Polyubiquitin-C [Cleaved into: Ubiquitin]	Control
Q0VCY1	VAPA_BOVIN	Vesicle-associated membrane protein-associated protein A (VAMP-A) (VAMP-associated protein A) (VAP-A)	Cytokine
Q6EMK4	VASN_HUMAN	Vasorin (Protein slit-like 2)	Both

Table S5. List of proteins classified into the category of *Structural/Adhesion/Junctional* proteins based on GO annotations

Accession	Entry name	Description	Sample
P60708	ACTB_HORSE	Actin, cytoplasmic 1 (Beta-actin) [Cleaved into: Actin, cytoplasmic 1, N-terminally processed]	Both
P63258	ACTG_BOVIN	Actin, cytoplasmic 2 (Gamma-actin) [Cleaved into: Actin, cytoplasmic 2, N-terminally processed]	Both
Q9BYX7	ACTBM_HUMAN	Putative beta-actin-like protein 3 (Kappa-actin) (POTE ankyrin domain family member K)	Control
Q3B7N2	ACTN1_BOVIN	Alpha-actinin-1 (Alpha-actinin cytoskeletal isoform) (F-actin cross-linking protein) (Non-muscle alpha-actinin-1)	Cytokine
O43707	ACTN4_HUMAN	Alpha-actinin-4 (Non-muscle alpha-actinin 4)	Cytokine
Q10741	ADA10_BOVIN	Disintegrin and metalloproteinase domain-containing protein 10 (ADAM 10) (EC 3.4.24.81) (Mammalian disintegrin-metalloprotease) (CD antigen CD156c)	Both
P78536	ADA17_HUMAN	Disintegrin and metalloproteinase domain-containing protein 17 (ADAM 17) (EC 3.4.24.86) (TNF-alpha convertase) (CD antigen CD156b)	Both
P04075	ALDOA_HUMAN	Fructose-bisphosphate aldolase A (EC 4.1.2.13) (Lung cancer antigen NY-LU-1) (Muscle-type aldolase)	Cytokine
Q9CZ52	ANTR1_MOUSE	Anthrax toxin receptor 1 (Tumor endothelial marker 8)	Both
Q8HZM6	ANXA1_HORSE	Annexin A1 (Annexin I) (Annexin-1) (Calpactin II) (Calpactin-2) (Lipocortin I)	Both
P84080	ARF1_BOVIN	ADP-ribosylation factor 1	Control
P62330	ARF6_HUMAN	ADP-ribosylation factor 6	Control
P18075	BMP7_HUMAN	Bone morphogenetic protein 7 (BMP-7) (Osteogenic protein 1) (OP-1) (Eptotermin alfa)	Cytokine
P55287	CAD11_HUMAN	Cadherin-11 (OSF-4) (Osteoblast cadherin) (OB-cadherin)	Both
Q3B7N0	CAD13_BOVIN	Cadherin-13	Both
Q9BY67	CADM1_HUMAN	Cell adhesion molecule 1 (Immunoglobulin superfamily member 4) (Nectin-like protein 2)	Both
P28491	CALR_PIG	Calreticulin (CRP55) (Calregulin) (Endoplasmic reticulum resident protein 60) (ERp60) (HACBP)	Both
P17655	CAN2_HUMAN	Calpain-2 catalytic subunit (EC 3.4.22.53) (Calcium-activated neutral proteinase 2) (CANP 2) (Calpain M-type)	Control
P40124	CAP1_MOUSE	Adenylyl cyclase-associated protein 1 (CAP 1)	Control
Q3V3V9	CARL2_MOUSE	Capping protein, Arp2/3 and myosin-I linker protein 2 (Capping protein regulator and myosin 1 linker 2)	Control
Q9BH13	CD166_BOVIN	CD166 antigen (Activated leukocyte cell adhesion molecule) (CD antigen CD166)	Both
Q05078	CD44_HORSE	CD44 antigen (Extracellular matrix receptor III) (GP90 lymphocyte homing/adhesion receptor) (Hyaluronate receptor)	Both
Q9N0K1	CD47_BOVIN	Leukocyte surface antigen CD47 (Integrin-associated protein) (IAP) (CD antigen CD47)	Both
P14209	CD99_HUMAN	CD99 antigen (12E7) (E2 antigen) (Protein MIC2) (T-cell surface glycoprotein E2) (CD antigen CD99)	Control
Q2KJ93	CDC42_BOVIN	Cell division control protein 42 homolog	Control
Q15517	CDSN_HUMAN	Corneodesmosin (S protein)	Control
Q14008	CKAP5_HUMAN	Cytoskeleton-associated protein 5 (Colonic and hepatic tumor overexpressed gene protein) (Ch-TOG)	Cytokine
Q8K4Q8	COL12_MOUSE	Collectin-12 (Collectin placenta protein 1) (CL-P1) (Scavenger receptor with C-type lectin)	Cytokine
Q7L576	CYFP1_HUMAN	Cytoplasmic FMR1-interacting protein 1 (Specifically Rac1-associated protein 1) (Sra-1) (p140sra-1)	Control
Q96F07	CYFP2_HUMAN	Cytoplasmic FMR1-interacting protein 2 (p53-inducible protein 121)	Control
P01040	CYTA_HUMAN	Cystatin-A (Cystatin-AS) (Stefin-A) [Cleaved into: Cystatin-A, N-terminally processed]	Both
P21291	CSRP1_HUMAN	Cysteine and glycine-rich protein 1 (Cysteine-rich protein 1) (CRP) (CRP1) (Epididymis luminal protein 141) (HEL-141)	Both
Q16832	DDR2_HUMAN	Discoidin domain-containing receptor 2 (Discoidin domain receptor 2) (EC 2.7.10.1) (CD167 antigen-like family member B) (CD antigen CD167b)	Both
P15924	DESP_HUMAN	Desmoplakin (DP) (250/210 kDa paraneoplastic pemphigus antigen)	Both
Q12959	DLG1_HUMAN	Disks large homolog 1 (Synapse-associated protein 97) (SAP-97) (SAP97) (hDlg)	Control

Table S5. (continued)

Accession	Entry name	Description	Sample
Q15700	DLG2_HUMAN	Disks large homolog 2 (Channel-associated protein of synapse-110) (Chapsyn-110) (Postsynaptic density protein PSD-93)	Control
Q14574	DSC3_HUMAN	Desmocollin-3 (Cadherin family member 3) (Desmocollin-4) (HT-CP)	Control
Q02413	DSG1_HUMAN	Desmoglein-1 (Cadherin family member 4) (Desmosomal glycoprotein 1) (DG1) (DGI) (Pemphigus foliaceus antigen)	Both
O43854	EDIL3_HUMAN	EGF-like repeat and discoidin I-like domain-containing protein 3 (Developmentally-regulated endothelial cell locus 1 protein) (Integrin-binding protein DEL1)	Both
P98172	EFNB1_HUMAN	Ephrin-B1 (EFL-3) (ELK ligand) (ELK-L) (EPH-related receptor tyrosine kinase ligand 2) (LERK-2)	Both
P00533	EGFR_HUMAN	Epidermal growth factor receptor (EC 2.7.10.1) (Proto-oncogene c-ErbB-1) (Receptor tyrosine-protein kinase erbB-1)	Both
P37176	EGLN_PIG	Endoglin (CD antigen CD105)	Control
Q9NZN4	EHD2_HUMAN	EH domain-containing protein 2 (PAST homolog 2)	Control
P14625	ENPL_HUMAN	Endoplasmin (94 kDa glucose-regulated protein) (GRP-94) (Heat shock protein 90 kDa beta member 1) (Tumor rejection antigen 1) (gp96 homolog)	Both
P29317	EPHA2_HUMAN	Ephrin type-A receptor 2 (EC 2.7.10.1) (Epithelial cell kinase) (Tyrosine-protein kinase receptor ECK)	Both
P54764	EPHA4_HUMAN	Ephrin type-A receptor 4 (EC 2.7.10.1) (EPH-like kinase 8) (EK8) (hEK8) (Tyrosine-protein kinase TYRO1) (Tyrosine-protein kinase receptor SEK)	Both
P54756	EPHA5_HUMAN	Ephrin type-A receptor 5 (EC 2.7.10.1) (Brain-specific kinase) (EPH homology kinase 1) (EHK-1) (EPH-like kinase 7) (EK7) (hEK7)	Control
O09127	EPHA8_MOUSE	Ephrin type-A receptor 8 (EC 2.7.10.1) (EPH- and ELK-related kinase) (Tyrosine-protein kinase receptor EEK)	Control
P54761	EPHB4_MOUSE	Ephrin type-B receptor 4 (EC 2.7.10.1) (Developmental kinase 2) (mDK-2) (Hepatoma transmembrane kinase) (Tyrosine kinase MYK-1)	Both
F1LYQ8	FARP1_RAT	FERM, ARHGEF and pleckstrin domain-containing protein 1 (FERM, RhoGEF and pleckstrin domain-containing protein 1)	Both
Q96AC1	FERM2_HUMAN	Fermitin family homolog 2 (Kindlin-2) (Mitogen-inducible gene 2 protein) (Pleckstrin homology domain-containing family C member 1)	Control
Q61851	FGFR3_MOUSE	Fibroblast growth factor receptor 3 (FGFR-3) (EC 2.7.10.1) (Heparin-binding growth factor receptor) (CD antigen CD333)	Control
Q5D862	FILA2_HUMAN	Filaggrin-2 (FLG-2) (Intermediate filament-associated and psoriasis-susceptibility protein) (Ifapsoriasin)	Both
P07589	FINC_BOVIN	Fibronectin (FN) [Cleaved into: Anastellin]	Both
P21333	FLNA_HUMAN	Filamin-A (FLN-A) (Actin-binding protein 280) (ABP-280) (Alpha-filamin) (Endothelial actin-binding protein) (Filamin-1) (Non-muscle filamin)	Both
O75369	FLNB_HUMAN	Filamin-B (FLN-B) (ABP-278) (ABP-280 homolog) (Actin-binding-like protein)	Cytokine
O43155	FLRT2_HUMAN	Leucine-rich repeat transmembrane protein FLRT2 (Fibronectin-like domain-containing leucine-rich transmembrane protein 2)	Both
P14207	FOLR2_HUMAN	Folate receptor beta (FR-beta) (Folate receptor 2) (Folate receptor, fetal/placental) (Placental folate-binding protein) (FBP)	Cytokine
Q9UP38	FZD1_HUMAN	Frizzled-1 (Fz-1) (hFz1) (FzE1)	Both
O75084	FZD7_HUMAN	Frizzled-7 (Fz-7) (hFz7) (FzE3)	Both
P32942	ICAM3_HUMAN	Intercellular adhesion molecule 3 (ICAM-3) (CDw50) (ICAM-R) (CD antigen CD50)	Control
P46940	IQGA1_HUMAN	Ras GTPase-activating-like protein IQGAP1 (p195)	Both
O14498	ISLR_HUMAN	Immunoglobulin superfamily containing leucine-rich repeat protein	Control
P56199	ITA1_HUMAN	Integrin alpha-1 (CD49 antigen-like family member A) (Laminin and collagen receptor) (VLA-1) (CD antigen CD49a)	Both
O75578	ITA10_HUMAN	Integrin alpha-10	Control
Q9UKX5	ITA11_HUMAN	Integrin alpha-11	Both
F1MMS9	ITA3_BOVIN	Integrin alpha-3 (CD49 antigen-like family member C) (Galactoprotein B3) (VLA-3 subunit alpha)	Both
Q27977	ITA5_BOVIN	Integrin alpha-5 (Fibronectin receptor subunit alpha) (Integrin alpha-F) (VLA-5)	Both
P23229	ITA6_HUMAN	Integrin alpha-6 (CD49 antigen-like family member F) (VLA-6) (CD antigen CD49f)	Both
P43406	ITAV_MOUSE	Integrin alpha-V (Vitronectin receptor subunit alpha) (CD antigen CD51) [Cleaved into: Integrin alpha-V heavy chain; Integrin alpha-V light chain]	Control
P53712	ITB1_BOVIN	Integrin beta-1 (Fibronectin receptor subunit beta) (VLA-4 subunit beta) (CD antigen CD29)	Both
P32592	ITB2_BOVIN	Integrin beta-2 (Cell surface adhesion glycoproteins LFA-1/CR3/p150,95 subunit beta) (Complement receptor C3 subunit beta) (CD antigen CD18)	Cytokine

Table S5. (continued)

Accession	Entry name	Description	Sample
P80747	ITB5_BOVIN	Integrin beta-5	Both
P26010	ITB7_HUMAN	Integrin beta-7 (Gut homing receptor beta subunit)	Both
Q96J84	KIRR1_HUMAN	Kin of IRRE-like protein 1 (Kin of irregular chiasm-like protein 1) (Nephrin-like protein 1)	Cytokine
Q769I5	MET_BOVIN	Hepatocyte growth factor receptor (HGF receptor) (EC 2.7.10.1)	Cytokine
Q95114	MFGM_BOVIN	Lactadherin (BP47) (Components 15/16) (MFGM) (MGP57/53) (Milk fat globule-EGF factor 8) (MFG-E8) (PAS-6/PAS-7 glycoprotein)	Both
O95297	MPZL1_HUMAN	Myelin protein zero-like protein 1 (Protein zero-related)	Both
Q148M6	MXRA8_BOVIN	Matrix remodeling-associated protein 8 (Limitrin)	Control
Q6VBQ5	MYADM_RAT	Myeloid-associated differentiation marker (Myeloid up-regulated protein)	Control
P35579	MYH9_HUMAN	Myosin-9 (Cellular myosin heavy chain, type A) (Myosin heavy chain 9) (Myosin heavy chain, non-muscle IIa) (Non-muscle myosin heavy chain A)	Control
Q9Y2A7	NCKP1_HUMAN	Nck-associated protein 1 (NAP 1) (Membrane-associated protein HEM-2) (p125Nap1)	Control
Q15223	NECT1_HUMAN	Nectin-1 (Herpes virus entry mediator C) (Nectin cell adhesion molecule 1) (Poliovirus receptor-related protein 1) (CD antigen CD111)	Both
Q9NQS3	NECT3_HUMAN	Nectin-3 (CDw113) (Nectin cell adhesion molecule 3) (CD antigen CD113)	Both
Q8NFZ4	NLGN2_HUMAN	Neuroligin-2	Control
Q04721	NOTC2_HUMAN	Neurogenic locus notch homolog protein 2 (Notch 2) (hN2)	Both
Q9Y639	NPTN_HUMAN	Neuroplastin (Stromal cell-derived receptor 1) (SDR-1)	Both
O14786	NRP1_HUMAN	Neuropilin-1 (Vascular endothelial cell growth factor 165 receptor) (CD antigen CD304)	Cytokine
O60462	NRP2_HUMAN	Neuropilin-2 (Vascular endothelial cell growth factor 165 receptor 2)	Both
O15018	PDZD2_HUMAN	PDZ domain-containing protein 2 (Activated in prostate cancer protein) (PDZ domain-containing protein 3)	Cytokine
P16234	PGFRA_HUMAN	Platelet-derived growth factor receptor alpha (PDGF-R-alpha) (PDGFR-alpha) (EC 2.7.10.1) (CD140a antigen)(Platelet-derived growth factor receptor 2)	Control
P09619	PGFRB_HUMAN	Platelet-derived growth factor receptor beta (PDGF-R-beta) (PDGFR-beta) (EC 2.7.10.1) (Platelet-derived growth factor receptor 1) (PDGFR-1) (CD antigen CD140b)	Both
Q92508	PIEZ1_HUMAN	Piezo-type mechanosensitive ion channel component 1 (Membrane protein induced by beta-amyloid treatment) (Mib) (Protein FAM38A)	Both
Q28161	PKP1_BOVIN	Plakophilin-1 (Band-6 protein) (B6P)	Cytokine
Q8SPJ1	PLAK_BOVIN	Junction plakoglobin (Desmoplakin III) (Desmoplakin-3)	Both
O14495	PLPP3_HUMAN	Phospholipid phosphatase 3 (EC 3.1.3.4) (Lipid phosphate phosphohydrolase 3) (PAP2-beta) (Phosphatidate phosphohydrolase type 2b)	Both
Q9UIW2	PLXA1_HUMAN	Plexin-A1 (Semaphorin receptor NOV)	Both
O43157	PLXB1_HUMAN	Plexin-B1 (Semaphorin receptor SEP)	Cytokine
O15031	PLXB2_HUMAN	Plexin-B2 (MM1)	Both
P07737	PROF1_HUMAN	Profilin-1 (Epididymis tissue protein Li 184a) (Profilin I)	Control
Q13308	PTK7_HUMAN	Inactive tyrosine-protein kinase 7 (Colon carcinoma kinase 4) (CCK-4) (Protein-tyrosine kinase 7) (Tyrosine-protein kinase-like 7)	Both
Q12913	PTPRJ_HUMAN	Receptor-type tyrosine-protein phosphatase eta (Protein-tyrosine phosphatase eta) (R-PTP-eta) (EC 3.1.3.48) (CD antigen CD148)	Control
P28828	PTPRM_MOUSE	Receptor-type tyrosine-protein phosphatase mu (Protein-tyrosine phosphatase mu) (R-PTP-mu) (EC 3.1.3.48)	Control
P62820	RAB1A_HUMAN	Ras-related protein Rab-1A (YPT1-related protein)	Both
P62998	RAC1_BOVIN	Ras-related C3 botulinum toxin substrate 1 (p21-Rac1)	Control
Q9Y6N7	ROBO1_HUMAN	Roundabout homolog 1 (Deleted in U twenty twenty) (H-Robo-1)	Both
P06702	S10A9_HUMAN	Protein S100-A9 (Calgranulin-B) (Calprotectin L1H subunit) (Leukocyte L1 complex heavy chain) (Migration inhibitory factor-related protein 14)	Cytokine
P60902	S10AA_BOVIN	Protein S100-A10 (Calpactin I light chain) (Calpactin-1 light chain) (Cellular ligand of annexin II) (S100 calcium-binding protein A10)	Control
Q58EX2	SDK2_HUMAN	Protein sidekick-2	Both

Table S5. (continued)

Accession	Entry name	Description	Sample
O00241	SIRB1_HUMAN	Signal-regulatory protein beta-1 (SIRP-beta-1) (CD172 antigen-like family member B) (CD antigen CD172b)	Both
Q71U36	TBA1A_HUMAN	Tubulin alpha-1A chain (Alpha-tubulin 3) (Tubulin B-alpha-1) (Tubulin alpha-3 chain) [Cleaved into: Detyrosinated tubulin alpha-1A chain]	Cytokine
Q13885	TBB2A_HUMAN	Tubulin beta-2A chain (Tubulin beta class IIa)	Control
Q9P273	TEN3_HUMAN	Teneurin-3 (Ten-3) (Protein Odd Oz/ten-m homolog 3) (Tenascin-M3) (Ten-m3) (Teneurin transmembrane protein 3)	Both
P04216	THY1_HUMAN	Thy-1 membrane glycoprotein (CDw90) (Thy-1 antigen) (CD antigen CD90)	Control
Q9Y490	TLN1_HUMAN	Talin-1	Both
Q13641	TPBG_HUMAN	Trophoblast glycoprotein (5T4 oncofetal antigen) (5T4 oncofetal trophoblast glycoprotein) (Wnt-activated inhibitory factor 1) (WAIF1)	Control
Q28178	TSP1_BOVIN	Thrombospondin-1 (Glycoprotein G)	Both
P30530	UFO_HUMAN	Tyrosine-protein kinase receptor UFO (EC 2.7.10.1) (AXL oncogene)	Both
Q28260	VCAM1_CANLF	Vascular cell adhesion protein 1 (V-CAM 1) (VCAM-1) (CD antigen CD106)	Both
P04004	VTNC_HUMAN	Vitronectin (VN) (S-protein) (Serum-spreading factor) (V75)	Control

Table S6. List of proteins classified into the category of *Extracellular matrix* proteins based on GO annotations

Accession	Entry name	Description	Sample
Q61072	ADAM9_MOUSE	Disintegrin and metalloproteinase domain-containing protein 9 (ADAM 9) (EC 3.4.24.-) (Meltrin-gamma) (Metalloprotease/disintegrin/cysteine-rich protein 9)	Both
P02461	CO3A1_HUMAN	Collagen alpha-1(III) chain	Both
Q3U962	CO5A2_MOUSE	Collagen alpha-2(V) chain	Cytokine
P12109	CO6A1_HUMAN	Collagen alpha-1(VI) chain	Both
P12110	CO6A2_HUMAN	Collagen alpha-2(VI) chain	Both
P12111	CO6A3_HUMAN	Collagen alpha-3(VI) chain	Control
A2AX52	CO6A4_MOUSE	Collagen alpha-4(VI) chain	Control
Q99715	COCA1_HUMAN	Collagen alpha-1(XII) chain	Cytokine
Q16832	DDR2_HUMAN	Discoidin domain-containing receptor 2 (Discoidin domain receptor 2) (EC 2.7.10.1) (CD167 antigen-like family member B) (CD antigen CD167b)	Both
P17813	EGLN_HUMAN	Endoglin (CD antigen CD105)	Both
P11276	FINC_MOUSE	Fibronectin (FN) [Cleaved into: Anastellin]	Control
P32942	ICAM3_HUMAN	Intercellular adhesion molecule 3 (ICAM-3) (CDw50) (ICAM-R) (CD antigen CD50)	Control
P11047	LAMC1_HUMAN	Laminin subunit gamma-1 (Laminin B2 chain)	Both
Q07954	LRP1_HUMAN	Prolow-density lipoprotein receptor-related protein 1 (LRP-1) (Alpha-2-macroglobulin receptor) (A2MR) (Apolipoprotein E receptor) (APOER) (CD antigen CD91)	Both
Q9GLE4	MMP14_BOVIN	Matrix metalloproteinase-14 (MMP-14) (EC 3.4.24.80) (Membrane-type matrix metalloproteinase 1) (MT-MMP 1)	Both
Q92626	PXDN_HUMAN	Peroxidasin homolog (EC 1.11.1.7) (Melanoma-associated antigen MG50) (Vascular peroxidase 1) (p53-responsive gene 2 protein)	Cytokine
O95980	RECK_HUMAN	Reversion-inducing cysteine-rich protein with Kazal motifs (hRECK) (Suppressor of tumorigenicity 15 protein)	Both
P04216	THY1_HUMAN	Thy-1 membrane glycoprotein (CDw90) (Thy-1 antigen) (CD antigen CD90)	Control
Q28178	TSP1_BOVIN	Thrombospondin-1 (Glycoprotein G)	Both
P04004	VTNC_HUMAN	Vitronectin (VN) (S-protein) (Serum-spreading factor) (V75) [Cleaved into: Vitronectin V65 subunit; Vitronectin V10 subunit; Somatomedin-B]	Control
