# Supplementary figures and images for: Alterations in the chondrocyte surfaceome in response to pro-inflammatory cytokines
Source: BMC Mol Cell Biol. 2020 Jun 26;21:47. doi: 10.1186/s12860-020-00288-9 (PMC7318434; doi:10.1186/s12860-020-00288-9)

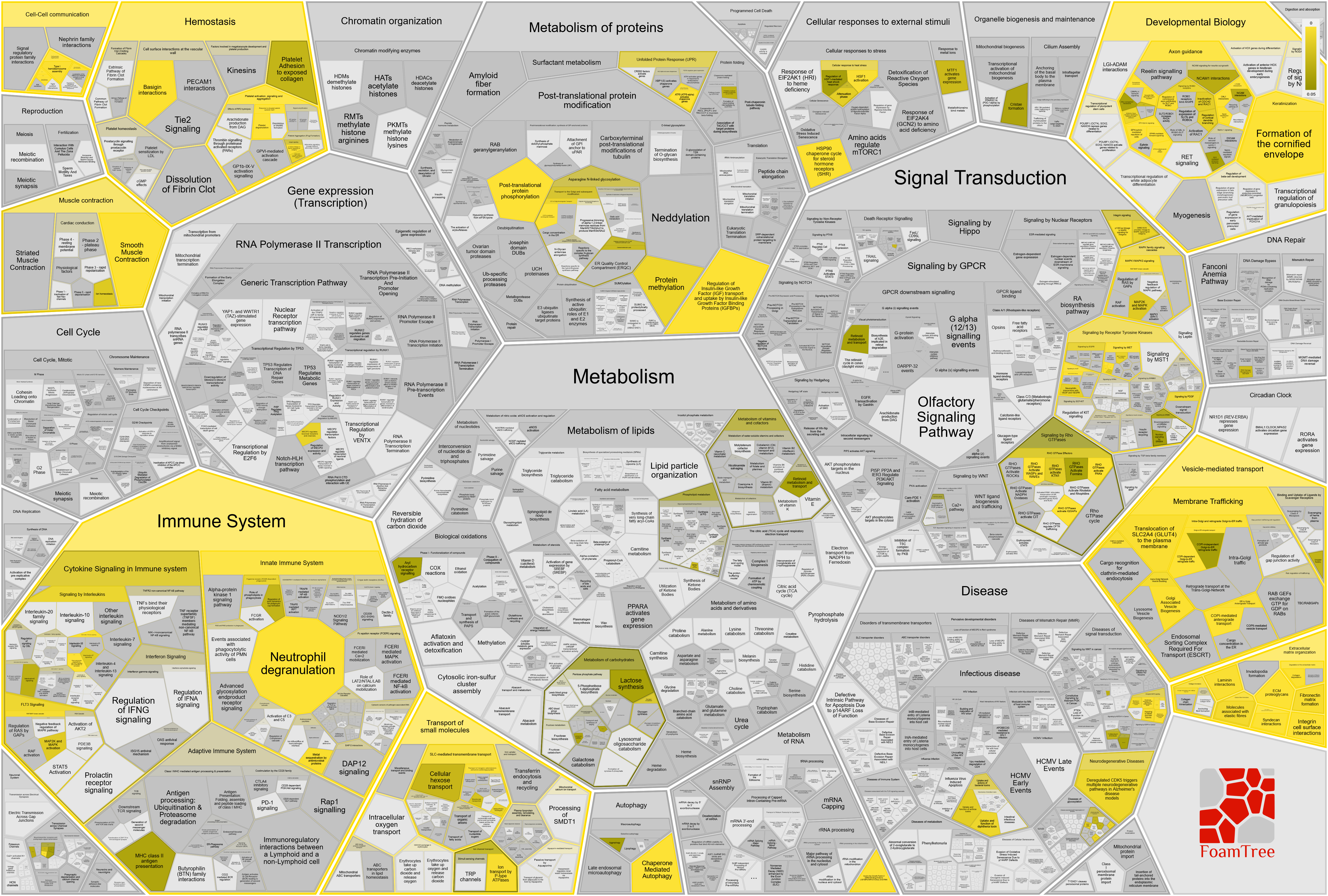

Supplement: Supplementary file 2 — Additional file 2. Foam tree of the over-represented pathways of the surface proteins identified in untreated control chondrocytes generated by the Reactome resource. [file 12860_2020_288_MOESM2_ESM.jpg]

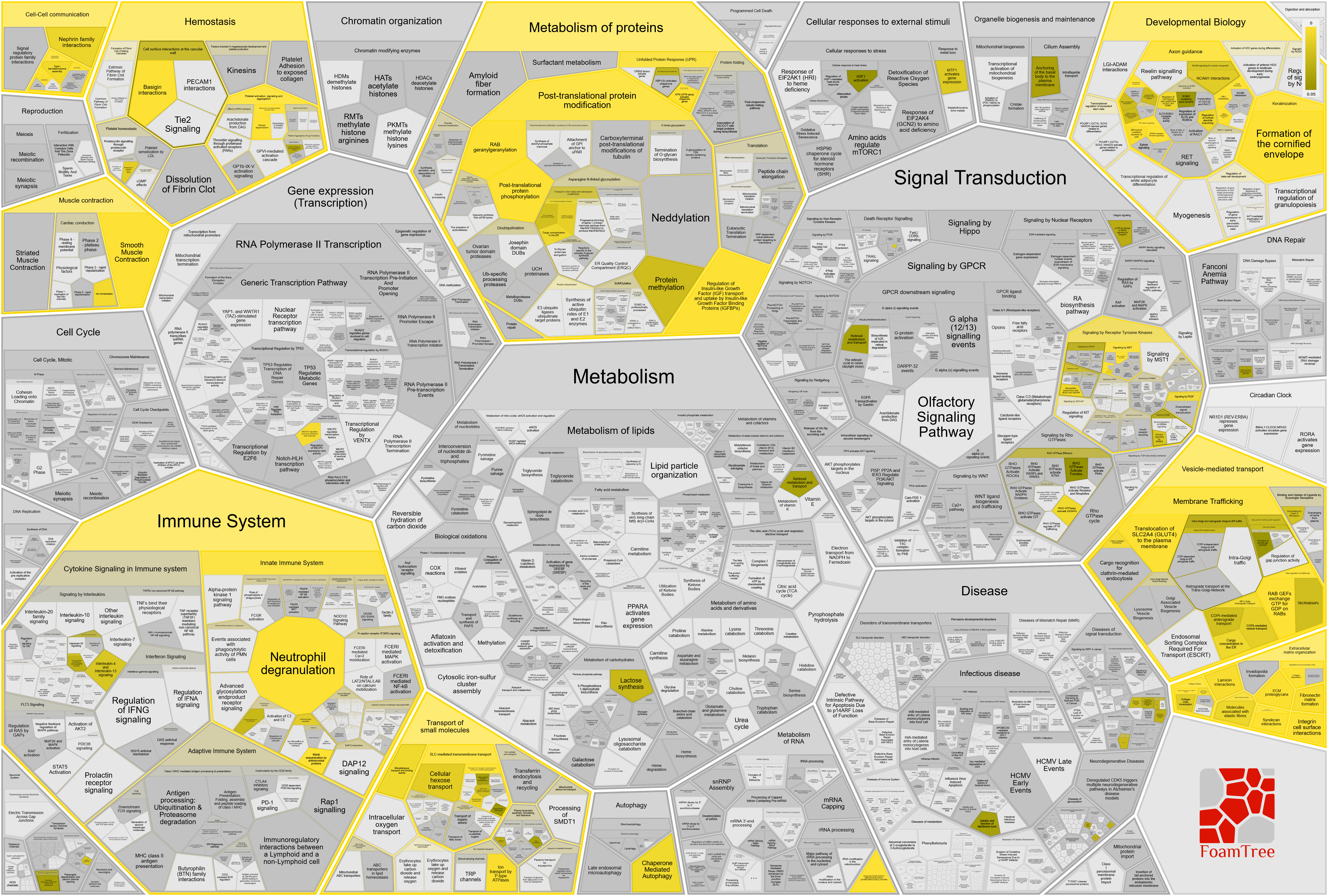

Supplement: Supplementary file 3 — Additional file 3. Foam tree of the over-represented pathways of the surface proteins identified in chondrocytes exposed to a pro-inflammatory micro-environment generated by the Reactome resource. [file 12860_2020_288_MOESM3_ESM.jpg]

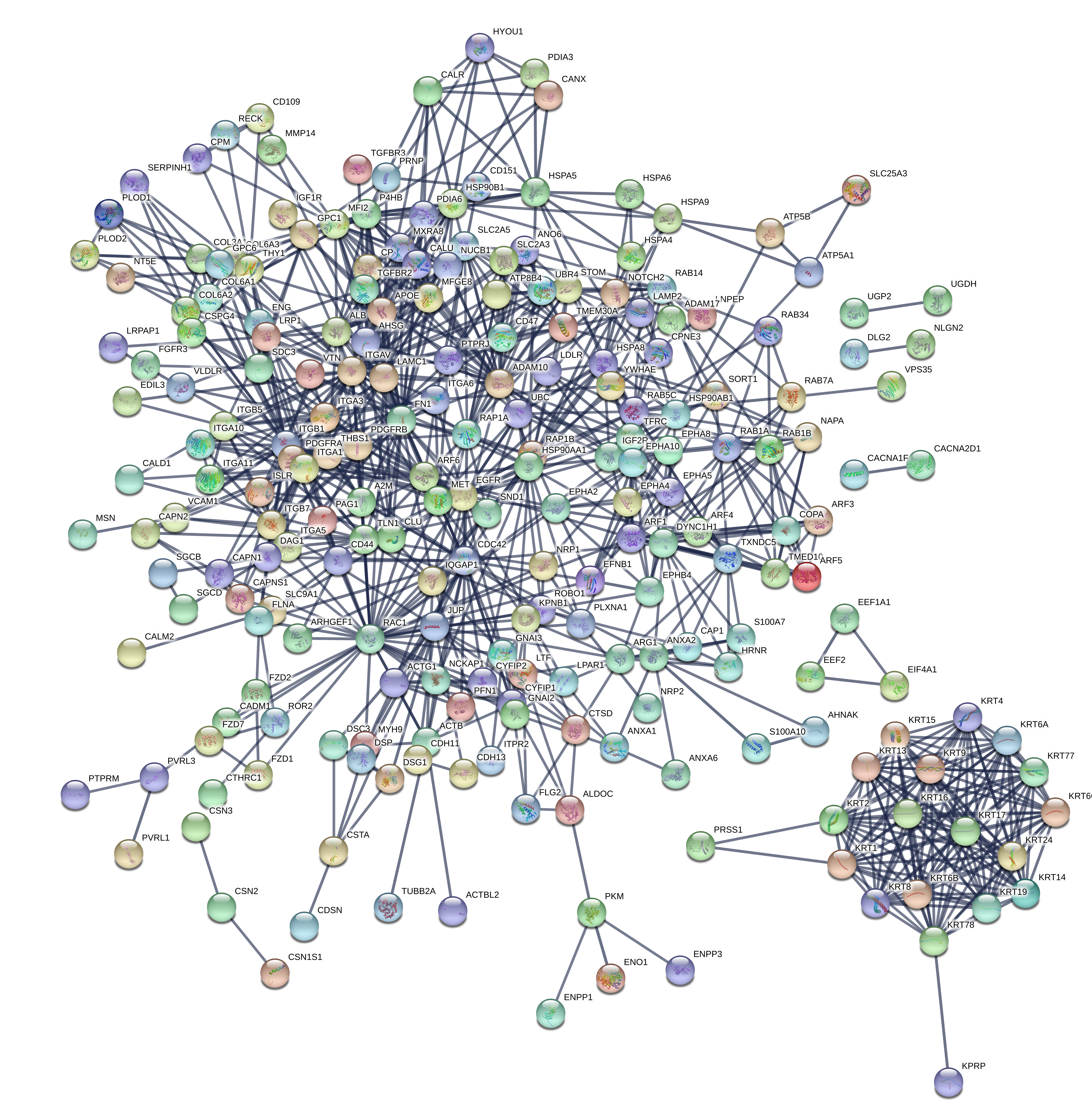

Supplement: Supplementary file 4 — Additional file 4. Predicted protein interactions in untreated control chondrocytes generated by the String resource. [file 12860_2020_288_MOESM4_ESM.png]

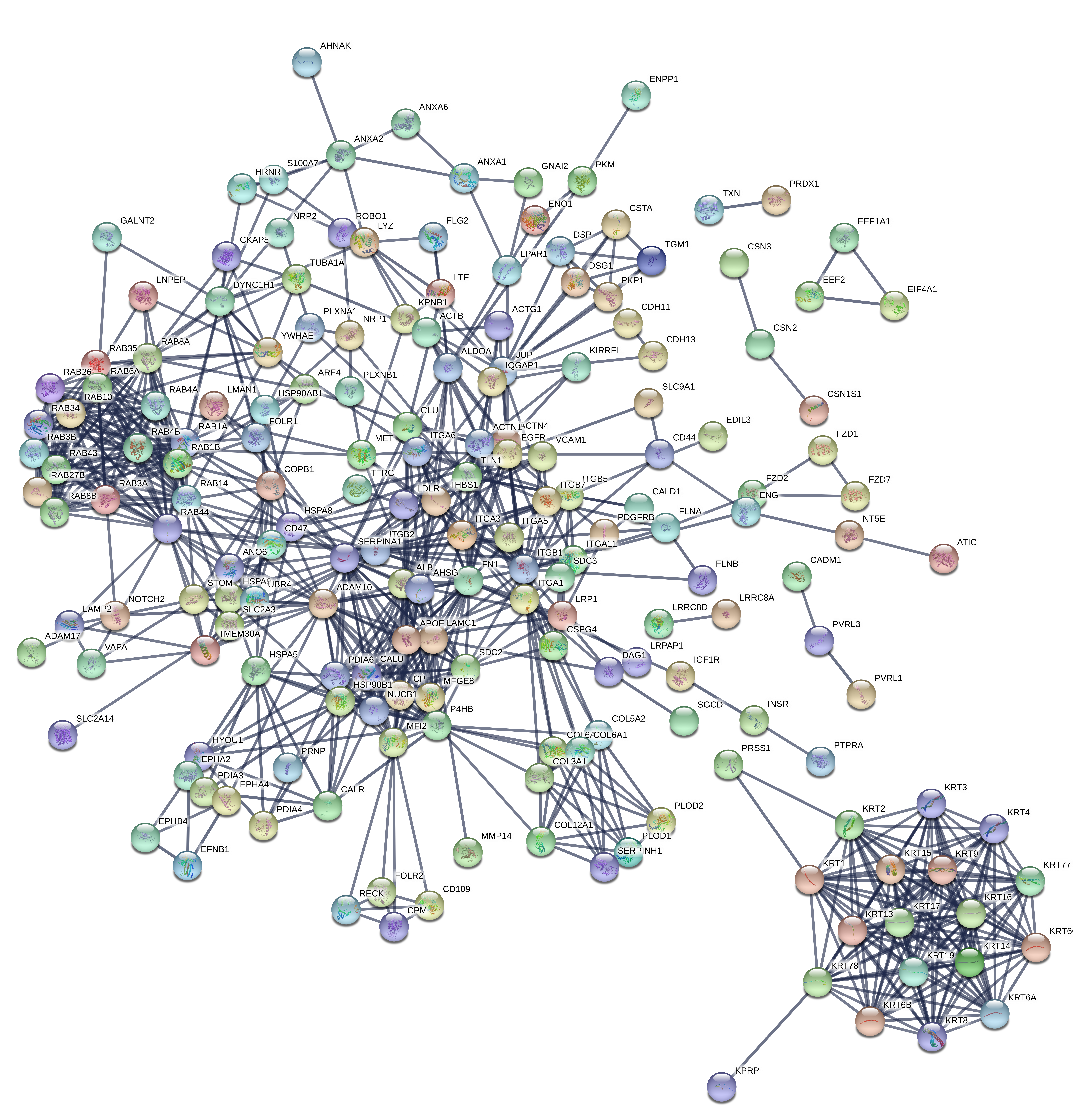

Supplement: Supplementary file 5 — Additional file 5. Predicted protein interactions in chondrocytes exposed to a pro-inflammatory micro-environment generated by the String resource. [file 12860_2020_288_MOESM5_ESM.png]
