# Supplementary material for: Alterations in the chondrocyte surfaceome in response to pro-inflammatory cytokines
Source: BMC Mol Cell Biol. 2020 Jun 26;21:47. doi: 10.1186/s12860-020-00288-9 (PMC7318434; doi:10.1186/s12860-020-00288-9)
Supplement: Supplementary file 7 — Additional file 7. This file contains the uncropped western blot membrane images presented in Figs. 2, 5 and 6. [file 12860_2020_288_MOESM7_ESM.pdf]

Supplementary Materials for

Alterations in the chondrocyte surfaceome in response to pro-inflammatory cytokines

Bernadette Jeremiasse, Csaba Matta*, Christopher R. Fellows, David J. Boocock, Julia R. Smith, Susan Liddell, Floris Lafeber, Willem E. van Spil, Ali Mobasheri

*Corresponding author. E-mail: matta.csaba@med.unideb.hu (C.M.)

This PDF file (Additional File #7) includes:

Figures S3–S12. Uncropped western blot membrane images

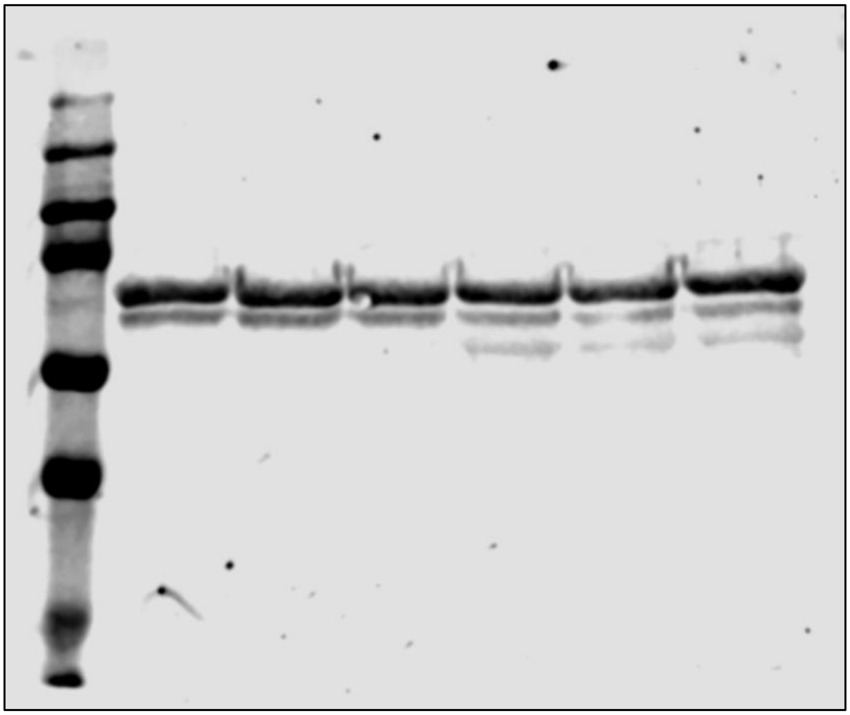

Figure S3. Uncropped western blot membrane image for the cropped bands shown in **Figure 2A**.

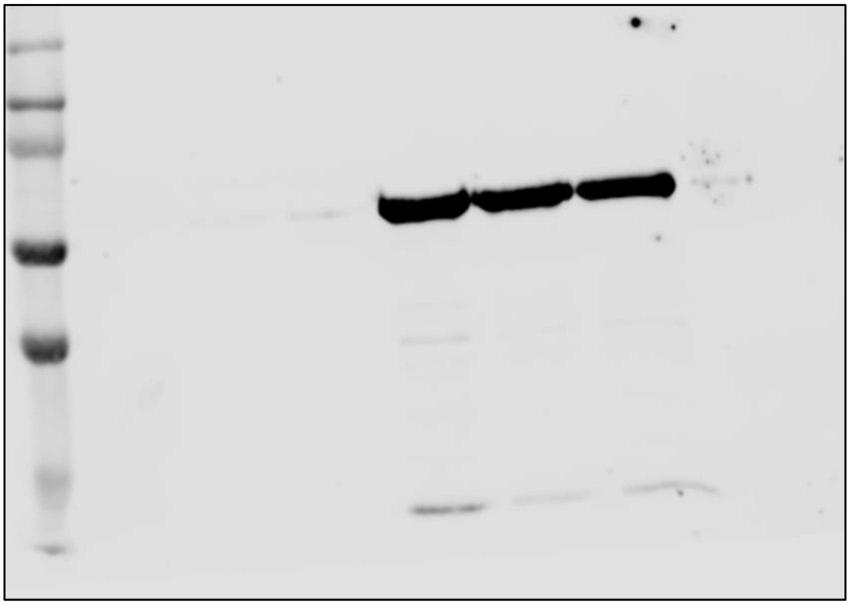

Figure S4. Uncropped western blot membrane image for the cropped bands shown in **Figure 2B**.

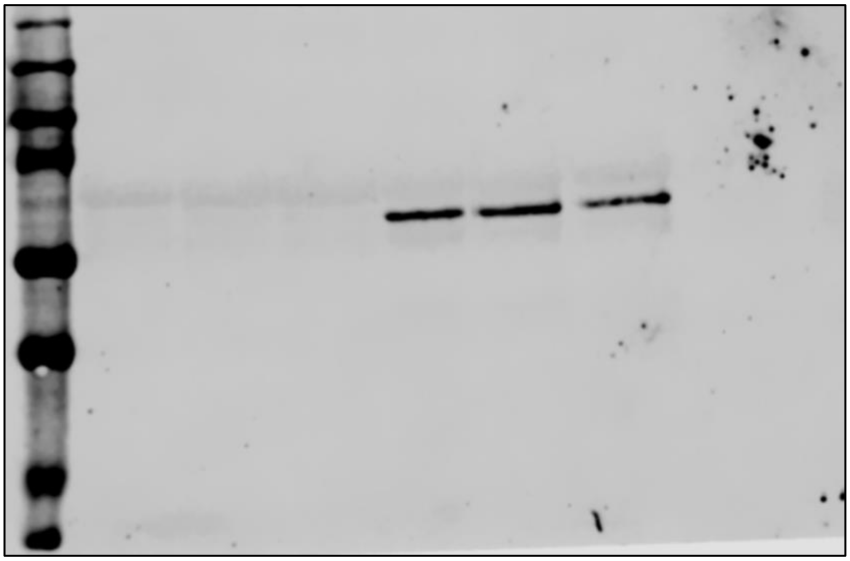

Figure S5. Uncropped western blot membrane image for the cropped bands shown in **Figure 2C**.

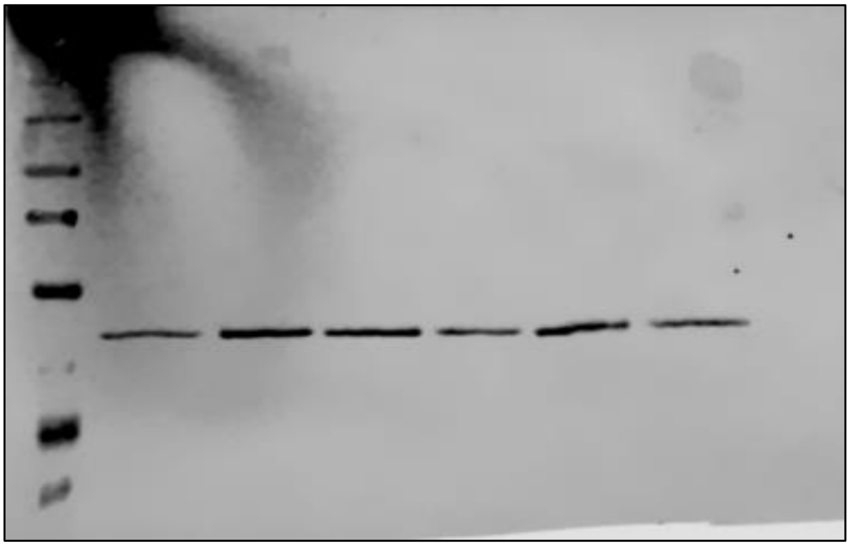

Figure S6. Uncropped western blot membrane image for the cropped bands shown in **Figure 2D**.

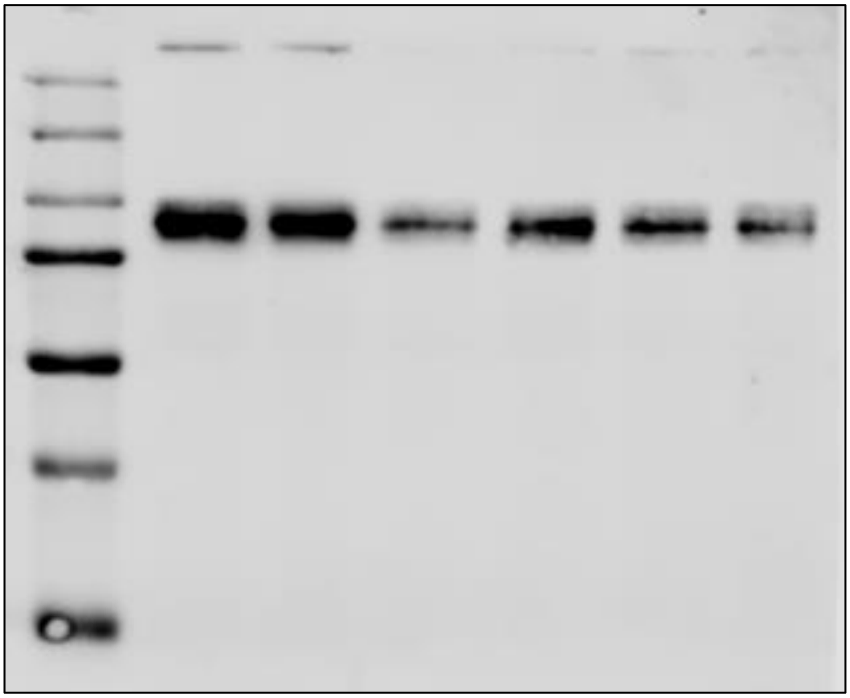

Figure S7. Uncropped western blot membrane image for the cropped bands shown in **Figure 5A**.

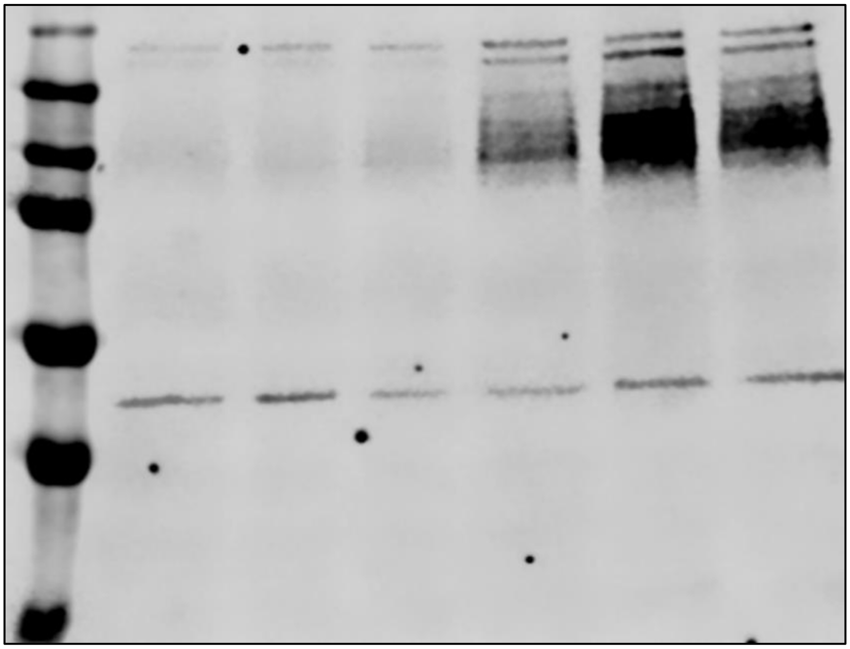

Figure S8. Uncropped western blot membrane image for the cropped bands shown in **Figure 5B**.

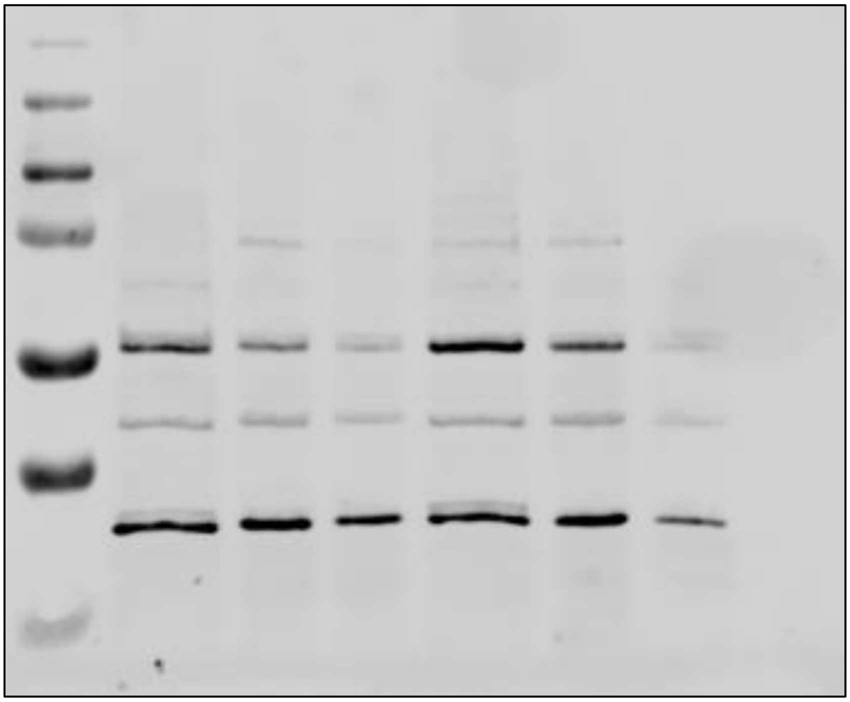

Figure S9. Uncropped western blot membrane image for the cropped bands shown in **Figure 5C** and **6A**.

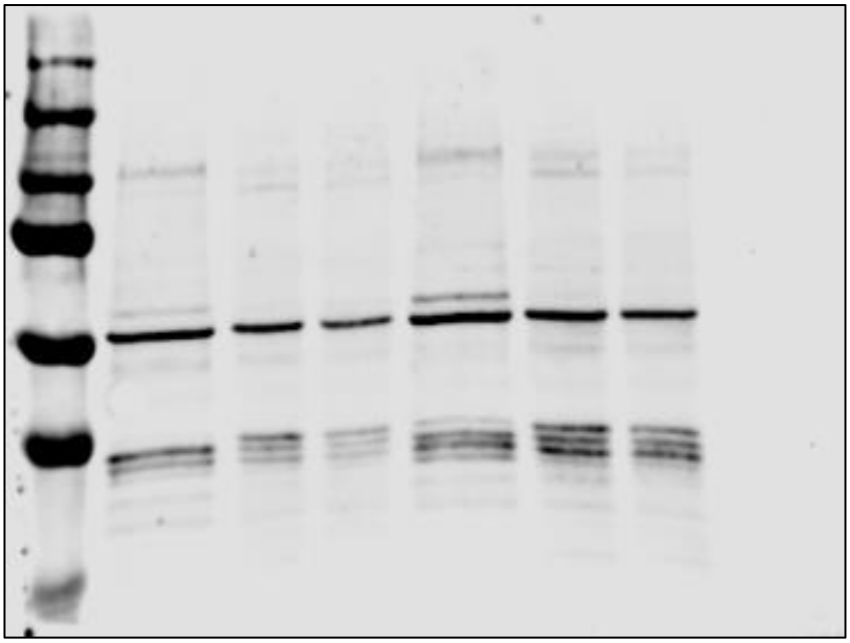

Figure S10. Uncropped western blot membrane image for the cropped bands shown in **Figure 5D** and **6B**.

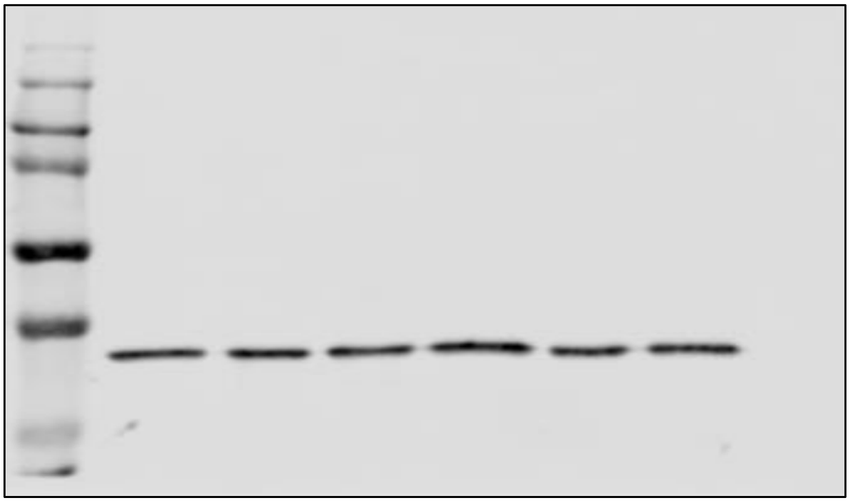

Figure S11. Uncropped western blot membrane image for the cropped bands shown in **Figure 5E**.

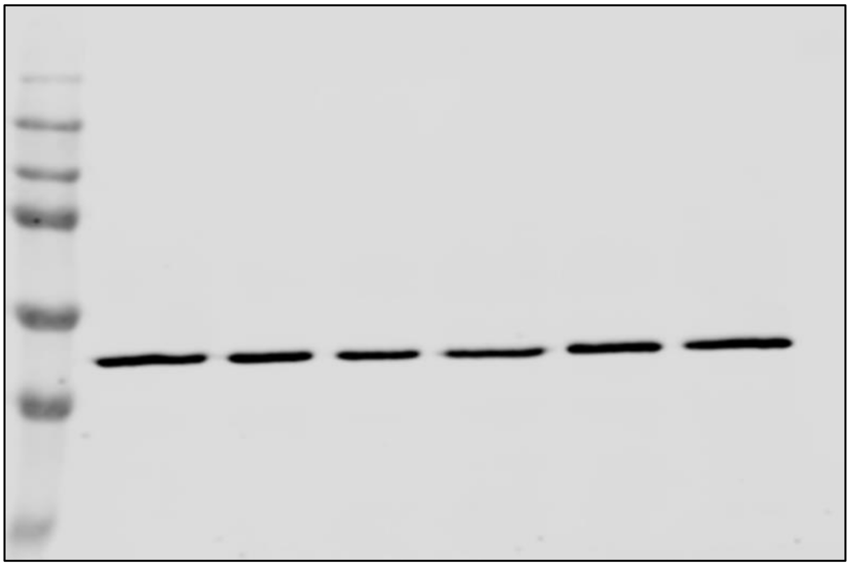

Figure S12. Uncropped western blot membrane image for the cropped bands shown in **Figure 5F**.
